# Stilbene derivative as a photosensitive compound to control the excitability of neonatal rat cardiomyocytes

**DOI:** 10.1042/BSR20181849

**Published:** 2019-01-25

**Authors:** Sheida R. Frolova, Vasili S. Gorbunov, Natalia S. Shubina, Alexander M. Perepukhov, Sandaara G. Romanova, Konstantin I. Agladze

**Affiliations:** Moscow Institute of Physics and Technology, Russian Federation, Dolgoprudny, Institutski lane, 9, Russia

**Keywords:** Excitation wave propagation, Neonatal rat cardiomyocyte, Stilbene derivative, voltage-gated channels

## Abstract

Substances that can be used as photosensitizers for cardiac tissue are very helpful in modeling various excitation patterns in a cardiac tissue culture and may have prospective use in the temporary and permanent ablation of unwanted excitation sources in the heart.

The aim of the present work is to study the effect of stilbene derivative c-TAB (2- {4- [(E) -2- (4-ethoxyphenyl) vinyl] phenoxy} ethyl) trimethylammonium bromide) on the cardiomyocyte layers and voltage-gated ion channels in cardiac cells. C-TAB is a structural analog to AzoTAB, reported previously as a photoswitch for cardiac and neural cells, in which the azobenzene moiety is replaced by a stilbene grouping. Such a replacement makes c-TAB less toxic to living cells. c-TAB has been shown to successfully inhibit excitation in cardiac cells in both *trans-* and *cis-* forms. The excitation inhibition of cardiac cells under c-TAB is reversible and can be overturned easily by washing out the c-TAB; however, not by light illumination. The irradiation of cardiac cells with near-UV, when the *trans-* form of c-TAB is applied, changes reversible inhibition to a permanent one that cannot be overturned by a washout.

## Introduction

A method, which allows photocontrol of the excitation waves in cardiac tissue, potentially, has two major fields of applications. First, controlling excitability of cardiac cells in a tissue culture in a patterned way provides a very convenient tool when studying excitation propagation in various conduction pathways and varying conditions. Second, it may have a prospective clinical use, for example, as a temporary and less damaging substitute for the ablation procedures, removing unwanted conduction circuits and excitation sources in a heart. Previously, we reported a method of photocontrol of cardiomyocyte layers with the aid of the azobenzene derivative AzoTAB (azobenzenetrimethylammonium bromide) [1,2]. By projecting computer-generated patterns on the AzoTAB-sensitized cardiomyocyte layers, it was possible to obtain temporary pathways for propagating excitation waves as well as for studying the behavior of cardiac reentry waves [3,4] in the tissue undergoing a decrease in excitability [3,4]. In that case, the sensitization of the cardiac cells to light is based on the *cis*- and *trans*- photoisomerization of the substance and the fact that *cis*- and *trans*- isomers of AzoTAB have different effects on the voltage-gated ion channels [5]. The present study showed that in its *trans*- form, AzoTAB suppresses fast Na^+^ and L-type Ca^2+^ currents and potentiates K^+^ currents. The transition of *trans*- AzoTAB to *cis*- AzoTAB obtained by UV irradiation leads to the recovery of normal channel functions [6,7]. Several azobenzene derivatives have been custom synthesized for photocontrol of neuronal activity. Among them are photoswitches that have been used by neuroscientists to modulate the activity of ion channels in neurons [6–8]. Maleimide-azobenzene-quaternary ammonium (Mal-Azo-QA), acrylamide-azobenzene-quaternary ammonium (AAQ) and benzoyl-azobenzene-quaternary ammonium (BzAQ) compounds suppress potassium channels K_v_. Quaternary ammonium-azobenzene-quaternaryammonium (QAQ) photosensitize not only K_v_, but also voltage-gated sodium (Na_v_) and calcium (Ca_v_) channels [9,10].

However, while AzoTAB reversibly enables the tuning of cardiomyocyte excitability to the desired degree [1,2], it is noticeably toxic to the cells. Thus, it has no pharmacological prospects and cannot be used in tissue culture experiments for extended periods of time (i.e. several hours or more).

**Figure 1:**
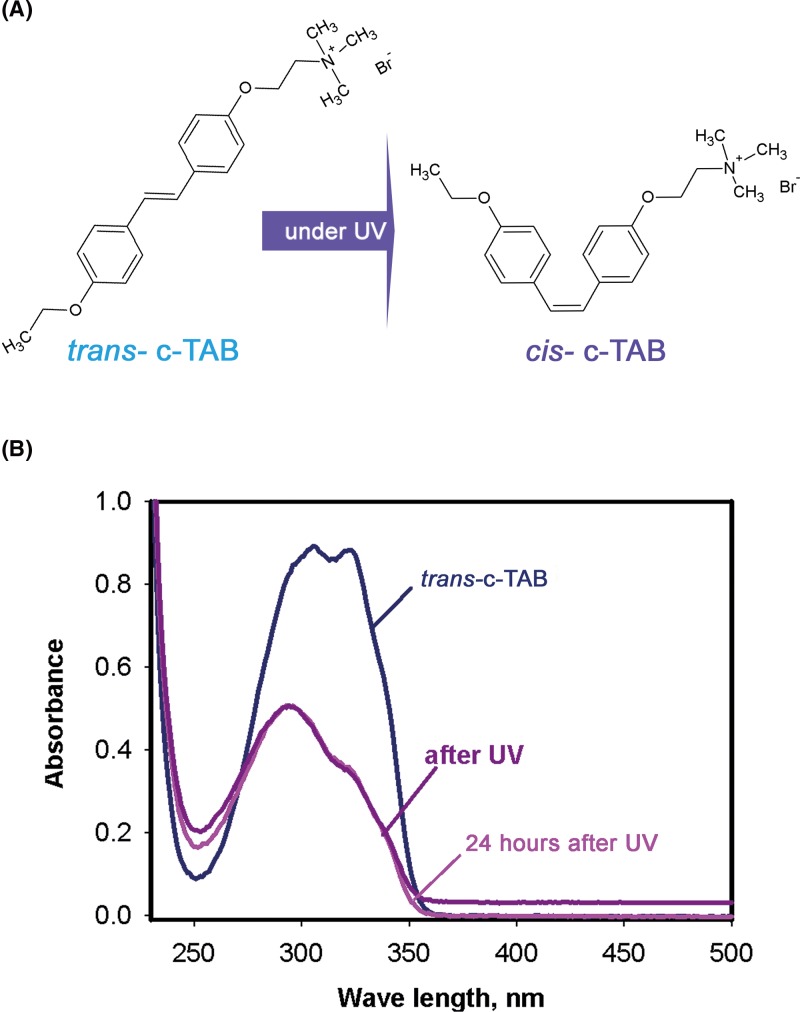
Photoisomerization of c-TAB (**A**) Schematic illustration of isomerization of c-TAB: *trans-* (left) and *cis*- (right) isomers of c-TAB. (**B**) Absorbance spectrum of 0.025 mM c-TAB in *trans*-form, after UV and 24 h after UV in Tyrode’s solution.

The present work is devoted to the study of the effect of stilbene derivative c-TAB (2- {4- [(E) -2- (4-ethoxyphenyl) vinyl] phenoxy} ethyl) trimethylammonium bromide) [11] on the cardiomyocyte layers and voltage-gated ion channels in cardiac cells. c-TAB is a structural analog to AzoTAB in which the azobenzene moiety is replaced by a stilbene grouping (Figure 1A). Such a replacement makes c-TAB less toxic to living cells. In our experiments, c-TAB was shown to successfully inhibit excitation in cardiac cells, as well as single cells and cells in a culture, in both, *trans-* and *cis-* forms. The excitation inhibition of cardiac cells under c-TAB is reversible and can be restored easily by washing out the c-TAB out; however, not by light illumination. Irradiation of cardiac cells with near-UV, when the *trans-* form of c-TAB is applied, changes reversible inhibition to a permanent one that cannot be overturned by a washout.

## Materials and methods

### Reagents

C-TAB was synthesized by Chem Rar corporation (Russian Federation) [11]. Amphotericine B and TEA were obtained from Wako Pure Chemical Industries, Ltd. (Japan).

Collagenase type II, penicillin/streptomycin, L-glutamine, and cell culture media Hank’s Balanced Salt Solution (HBSS) and Dulbecco’s modified Eagle's medium (DMEM), L15, Human plasma fibronectin, MTT assay kit were purchased from Gibco I Thermo Fisher Scientific Inc. (U.S.A.). Other chemical reagents were obtained from Helicon (Russian Federation).

### Preparation of neonatal rat ventricular cardiomyocyte (NRVM) for patch clamp for optical mapping

Hearts were isolated from neonatal 1–2-day-old Wistar rats using a two-day protocol of Neonatal Cardiomyocyte Isolation System of Worthington Biochemical Corporation [12] with some modifications. We do not use trypsin inhibitor on the second day, since minced neonatal hearts are washed out from trypsin and this efficiently stops digesting. The second day of isolation, we conduct at room temperature. Briefly, neonatal hearts were minced, stayed with trypsin 16 h and dispersed in a collagenase type 2 solution. To remove the fibroblasts after centrifugation, the isolated cells were suspended in Dulbecco’s modified Eagle’s medium (with 10% fetal bovine serum, 1% penicillin streptomycin, and 1% kanamycin) and kept in a tissue culture dish for 1 h. Non-adherent cells were collected and plated on a glass based dish (27-mm j glass window) coated with human plasma fibronectin (Gibco) and incubated at 37°C under humidified 5% CO_2_ conditions for 1 day. After incubation, the medium was replaced with a minimum essential medium containing 10% calf serum, 1% penicillin-streptomycin and 1% kanamycin; and the incubation continued for 5 to 8 days until confluent monolayers were formed [5].

#### Monitoring and control of excitation

The method of monitoring the excitation waves have been described in our previous publications [1,2]. Excitation waves were monitored by using the Ca^2+^ - sensitive fluorescent dye Fluo-4 (Invitrogen). The dye was added at a final concentration of 10 mM into standard Tyrode’s solution (Sigma) for 60 min before the onset of the experiments. Images were acquired using a CMOS camera (pco.1200hs; PCO AG) equipped with an image intensifier unit (C8600; Hamamatsu) and connected to a macro-view MVX10 Olympus microscope. Data were acquired at 50 frames per second using 480 × 480-pixel resolution. The fluorescent dye was excited at *λ* = 490 nm using the microscope’s light source unit outfitted with a mercury lamp and a blue bandpass filter. The same blue light source was used to induce the *trans*- conformation of c-TAB. To induce the *trans*- to *cis*- transition, we utilized a 365-nm UV-LED module (LC-L2; Hamamatsu). The power densities of blue and UV lights at the culture level were 3.5 and 7 mW/cm^2^, respectively. The images were analyzed by using an ImageJ image processing system (NIH). A time–space plot was made by the Reslice function of ImageJ with horizontal 1 pixel at 10 ms. In several experiments, we used electrical stimulation to induce an excitation wave. Rectangular pulses with amplitudes ranging from 1.5 to 4.0 V and duration of 20 ms were delivered via a 1-mm non-polarizing platinum electrode [1].

### Preparation of neonatal rat ventricular cardiomyocyte (NRVM) for patch clamp

Wistar rats were purchased from branch “Andreevka” of Federal State Budgetary Science Institution “Scientific Center for Biomedical Technologies” of the Federal Medical and Biological Agency. Isoflurane was used as an anesthetic for pups.

All the experiments conformed to the Guide for the Care and Use of Laboratory Animals, published by the United States National Institutes of Health (Publication No/ 85-23, revised 1996) and approved by the Moscow Institute of Physics and Technology Life Science Center Provisional Animal Care and Research Procedures Committee, Protocol #A2-2012-09-02. All applicable international, national, and/or institutional guidelines for the care and use of animals were followed.

Cardioectomy was performed on 1–2-day-old neonatal rat pups under isoflurane anesthesia, followed by enzymatic dissociation of cardiomyocytes as described in detail in Neonatal Cardiomyocyte Isolation System of Worthington Biochemical Corporation [12]. The isolated cardiac cells were plated at low density (∼2.8 × 10^4^ cells/cm^2^) onto fibronectin-coated microscope coverslips for patch-clamp experiments. The cells were incubated in DMEM, supplemented with 10% FBS, 2 mM of L-glutamine and 100 U/ml of penicillin/streptomycin at 37°C in 5% CO^2^. Twenty-four hours after isolation and plating, the cells were washed with warm PBS and cultured in DMEM with 5% FBS as described in our previous publication [5].

#### Electrophysiology

Whole-cell currents were recorded, similar as described previously [5], in single cardiomyocytes, which were unconnected to neighboring cells, using the perforated patch-clamp technique. As a perforating agent Amphotericine B was used at a final concentration of 0.24 mg/ml [13]. The experiments were carried out at room temperature (22-24°C) on days 1 through 3 postplating.

A coverslip with cultured cardiac cells was placed in a recording chamber mounted on the stage of an Olympus IX71 inverted microscope table. The chamber was continuously perfused with an appropriate bathing solution.

#### Recording solutions

The bathing solution used for recording Nav and Ca, *L-type* currents contained 10 mM HEPES/NaOH, 80 mM NaCl, 20 mM TEA-Cl, 10 mM CsCl, 1.2 mM KH_2_PO_4_, 5 mM MgSO_4_, 2 mM CaCl_2_, 20 mM D-glucose, pH 7.25 (270 mOsm). The pipette solution contained 10 mM HEPES/NaOH, 130 mM CsCl, 5 mM MgSO_4_, 5 mM EGTA, pH 7.25 (285 mOsm). For recording INav, 0.001 mM Nifedipine was added to bathing solution separately.

For the whole-cell recording of Kv currents, the bathing solution contained 10 mM HEPES/KOH, 80 mM NaCl, 5 mM KCl, 1.2 mM KH_2_PO_4_, 5 mM MgSO_4_, 2 mM CaCl_2_, 20 mM D-Glucose, pH = 7.25 (270 mOsm), and the patch pipette was filled with a solution containing 10 mM HEPES/KOH, 130 mM KCl, 5 mM MgSO_4_, 5 mM EGTA, pH = 7.25 (285 mOsm) [5]. For Ito the bathing solution contained 143 mM NMDG, 5 mM HEPES/KOH, 5.4 mM KCl, 0.5 mM MgCl_2_, 1.8 mM CaCl_2_, 10 mM D-Glucose, 0.001 mM Nifedipine, pH 7.2. The pipette solution contained 135 mM KCl, 5 mM NMDG, 10 mM HEPES/KOH, 5 mM EGTA, 5 mM M gATP, pH 7.2. The bathing solution used for recording the action potential contained 150 mM NaCl, 5.4 mM KCl, 1.8 mM CaCl_2_, 1 mM MgSO_4_, 15 mM D-glucose, 15 mM HEPES/NaOH and 1 mM Na-pyruvate at a pH 7.4; the pipette solution contained 150 mM KCl, 5 mM NaCl, 2 mM CaCl_2_, 5 mM EGTA, 10 mM HEPES/KOH and 5 mM MgATP at a pH 7.25.

A 5 mM stock solution of c-TAB was prepared in DMSO and stored at room temperature with protection from light. For electrophysiological measurements, c-TAB at a final concentration of 60 μM was used and it is added directly to the recording chamber, as required. The cardiomyocytes were pre-equilibrated in the c-TAB-containing solution for at least 3 min before electrical stimulation sequences were begun.

#### Voltage clamp experiments

Patch pipettes were pulled from borosilicate glass (BF150-86-10 Sutter Instrument, U.S.A.) with tip resistances of ∼3 MΩ when placed into the experimental solution. The pipette offset was corrected to zero just prior to the formation of a gigaohm (GΩ) seal. After the formation of GΩ seal, the pipette capacitance was cancelled using the amplifier fast capacitance cancellation settings. Electrical access to the cell by perforation was indicated by the appearance of slow capacitance currents that increased the amplitude and rate of decay when more amphotericin pores formed in the membrane enclosed by the patch pipette.

#### Ionic currents

Peak Ca^2+^ currents, steady-state K^+^ currents and Na^+^ currents generated in isolated cardiomyocytes were compared before and ∼3 min after the addition of 60 µM c-TAB, as well as upon subsequent irradiation with near-UV light.

The effect of c-TAB on whole-cell currents evoked by ramping up stimuli from −120 mV to +50 mV was examined over a 200-ms period, with a holding potential (HP) of −80 mV (using a prestep: −80 mV to −120 mV for 100 ms) [14]. The changes in the ramp currents in the absence and presence of *trans*- c-TAB, as well as after UV irradiation (*λ* ∼ 365 nm), were directly compared in a single cardiomyocyte. The percent inhibition was calculated by dividing the ramp peak recorded after treatment with 60 µM *trans*- c-TAB by the ramp peak of the control.

In similar experiments conducted with I_Ca_, *_L_*, the effect of c-TAB on cardiac L-type Ca^2+^ currents was examined in neonatal rat ventricular cardiomyocytes (NRVM). The effect of c-TAB on I_Ca_, *_L_* was analyzed using CsCl-rich solutions and TEA^+^ to suppress K^+^ currents. To study L-type Ca^2+^ currents without contamination from Na^+^ currents, a 100-ms prepulse to −40 mV from a HP of −80 mV was used [15,16]. The peak I_Ca_, *_L_* was measured at 0 mV. Outward IKs was elicited by a 500-ms depolarizing pulse from 0 mV to +60 mV (HP of −70 mV). The amplitude of the IK was measured at the end of the voltage step. Ito was measured by voltage steps from −50 mV to +60 in 10 mV increments (400 ms duration) [5].

The action potentials (APs) were elicited by 4 ms-long current injections (1 nA) with frequency 1 Hz, and the strength of the pulse was increased stepwise until a stable action potential with a peak above the 0-mV line was reached.

Typically, the membrane capacitances measured with pCLAMP10.2 software ranged from 5 to 40 pF.

### Photoisomerization of c-TAB

Photoisomerization of *trans*- c-TAB to a *cis-* isomer was induced by illuminating with ∼365 nm near-UV light for 90 s, according to our established methodology [1]. A UV-LED (Model: LC-L1V3, Hamamatsu, Japan) was used as a light source. The light was applied to the experimental chamber using an LED head unit (Series: L11921, Hamamatsu, Japan). The power density of the UV light was ∼170 mW/cm^2^ and was measured with a laser power meter Nova II P/N 7Z01550 (Ophir Spiricon Europe GmgH, Darmstadt, Germany) at the end of the LED head unit.

### Spectral measurements

The absorption spectra of c-TAB isomers were recorded in Tyrode’s solution using a spectrophotometer (U0080D; Hitachi). Before spectra recordings, the samples were illuminated for 1 min using either 490 nm blue or 365 nm UV light.

### NMR measurements

NMR spectra were recorded with the aid of Varian Inova 500 WB (with the resonance frequency for nuclei ^1^H 500 МHz) at room temperature.

### Cytotoxicity analysis

Cytotoxicity was measured by MTT assay [17] on neonatal rat ventricular myocytes (NRVM) at 120 min of incubation in c-TAB and azoTAB.

### Data analysis

The data analysis was performed using Clampfit 10.2 (Molecular Devices, U.S.A.) and SigmaPlot 12.0 (Systat Software, U.S.A.) software. Numerical values are given as the mean ± SEM and averaged from at least three neonatal cardiomyocytes from independent cell cultures. Statistical significance was evaluated by the Student's *t*-test, with a value of *P*<0.05.

## Results

Similar to AzoTAB, с-TAB has two isoforms, which are shown schematically in Figure 1A. The absorption spectrum of the *trans*- isomer (blue line) is in the 320-nm region (Figure 1A). Irradiation with this wavelength leads to a *trans*- *cis* transition. Figure 1B shows the absorption spectra of *trans*- and *cis*- isomers of c-TAB, as well as the absorption spectrum of c-TAB 24 h after its irradiation with UV. It can be seen that there is no thermal relaxation of the *cis*- c-TAB to its *trans*- form. In addition, unlike AzoTAB, the *cis*- form of c-TAB cannot be transformed to a *trans*- isomer by light.

**Figure 2:**
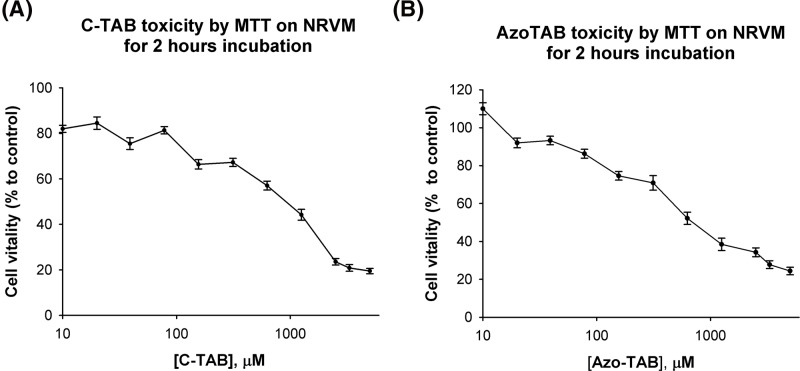
The cytotoxicity analysis by MTT assay protocol (**A**) The cytotoxicity of c-TAB. The cytotoxicity of c-TAB was carried by MTT test. The sample number of cells to each point is more than 3 (*n*>3). (**B**) The cytotoxicity of AzoTAB. The cytotoxicity of Azo-TAB was carried by MTT test. The sample number of cells to each point is more than 3 (*n*>3).

A lower cytotoxicity of c-TAB compared with AzoTAB is demonstrated in Figure 2. The IC50 value of the c-TAB on NRVM is 0.9 mM and for the AzoTAB is 0.7 mM.

### Suppression of excitation waves in the NRVM layer by c-TAB

**Figure 3:**
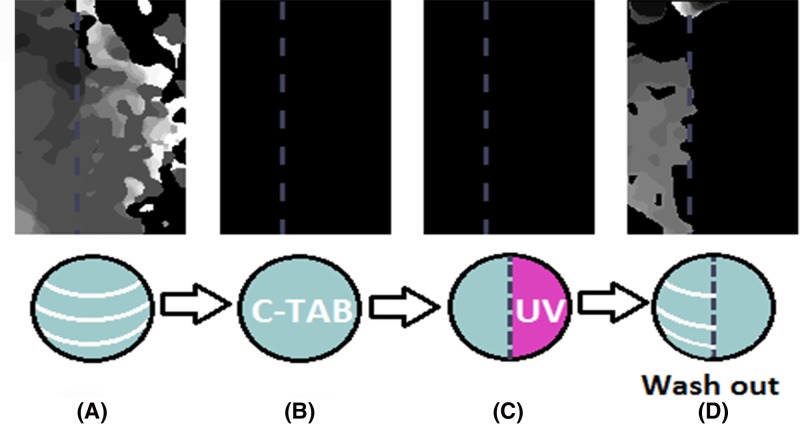
Effect of *trans-* c-TAB on the generation and propagation of excitation waves Activation maps of excitable NRVM monolayer: (**A**) control of excitable NRVM monolayer; (**B**) addition of 50 µM of c-TAB to control the excitable NRVM monolayer. The excitation of the NRVM monolayer was suppressed. Similar results were obtained in three additional samples of NRVM monolayers. (**C**) The right part of the monolayer was irradiated by UV light for 1 min. The excitation of the NRVM monolayer was not restored. (**D**) Excitability was restored after the c-TAB was washed out of the of NRVM monolayer in the half of the sample that had not been previously illuminated.

Figure 3 (S1_movie–S4_movie in Supplementary Materials) shows the suppression of excitation waves in a layer of NRVM cells in the presence of *trans*- c-TAB. c-TAB was added to the culture medium to a concentration of 50 μM. After 5 min, the propagation of excitation waves was blocked completely (Figure 3B). Then, the right part of the slide with the cells was illuminated with UV at an intensity of 5 mW/cm^2^ for 1 min. Unlike the case of AzoTAB, there were no observed excitation waves in the illuminated region, i.e., the electrical conductivity block remained after the UV-induced *trans*–*cis* isomerization (Figure 3C). The restoration of blocked excitation waves in NRVM became possible after the subsequent washing of *trans*- c-TAB from the cells (Figure 3D). In this case, the excitation wave velocity is restored up to 80% of the initial level.

**Figure 4:**
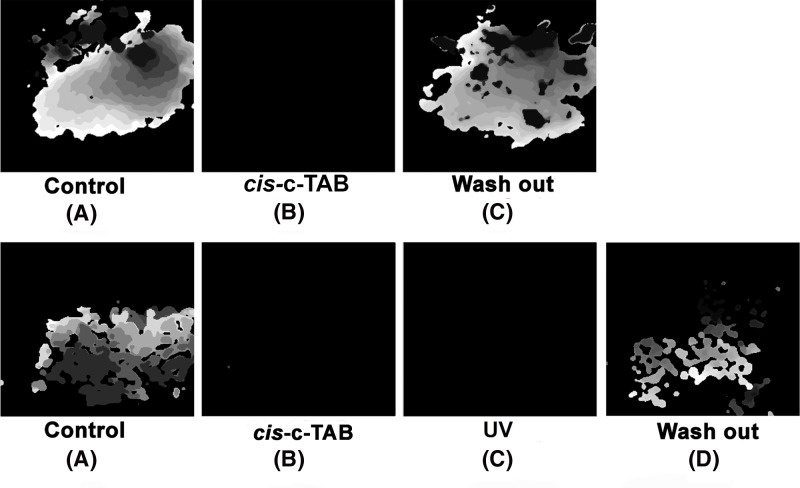
Effect of *cis*- c-TAB on NRVM monolayer Activation maps of excitable NRVM monolayer: Top line: (**A**) Activation maps of excitable NRVM monolayer (control). (**B**) Addition of 50 µM of cis- c-TAB leads to suppression of the excitation of the NRVM monolayer. (**C**) Activation maps of the excitable NRVM monolayer after washout of *cis*- c-TAB. The present study showed that the excitation of the NRVM monolayer was restored. Similar results were obtained in three additional samples of NRVM monolayers. Bottom line: (**A**) Activation maps of excitable NRVM monolayer (control). (**B**) Addition of 50 µM*cis*- c-TAB leads to suppression of the excitation of the NRVM monolayer. (**C**) Activation maps of the NRVM monolayer after UV irradiation of the NRVM monolayer in the presence of *cis*- c-TAB. (**D**) Activation maps of the excitable NRVM monolayer after washout of *cis*- c-TAB. The present study showed that the excitation of the NRVM monolayer was restored. Similar results were obtained in three additional samples of NRVM monolayers.

Figure 4 (S5_movie–S11_movie in Supplementary Materials) shows the suppression of excitation waves in a layer of NRVM cells in the presence of *cis*- c-TAB that was previously UV-irradiated and then added to cells. When the previously UV-irradiated c-TAB (*cis*- c-TAB) was added to the cell culture in the *cis*- form, a similar suppression of the excitation waves was observed (Figure 4B). However, a recovery of the blocked excitation waves in the NRVM appeared possible by the subsequent washing of *cis*- c-TAB (which was pre-irradiated and then added to the cells) from the cells (Figure 4, top line). In this case, the recovery of the speed of the excitation wave is possible up to 70% of the initial level. The present study showed that even after UV irradiation of the NVRM monolayer in the presence of *cis*- c-TAB (which was pre-irradiated and then added to the cells), it is possible to restore excitation waves by washing (Figure 4, bottom line and Table 1).

**Table 1:** Effect of stilbene derivative c-TAB in various conditions on layer of NRVM

NVRCM monolayer	Under *trans*-c-TAB	Washout of *trans*- c-TAB	UV illumination under *trans*-c-TAB	Washout after UV illumination under *trans*- c-TAB	Under *cis*-c-TAB	Washout of *cis*- c-TAB	Washout after UV illumination under *cis*- c-TAB
Propagation of excitation waves	Blocked	Recovered	Blocked	Blocked	Blocked	Recovered	Recovered

Thus, both the *trans*- and *cis*- isomers of c-TAB inhibited the origination and propagation of excitation waves in the NRVM monolayers. Unlike the case of AzoTAB, it was not possible to tune the excitability of the cell layer with light only.

**Figure 5:**
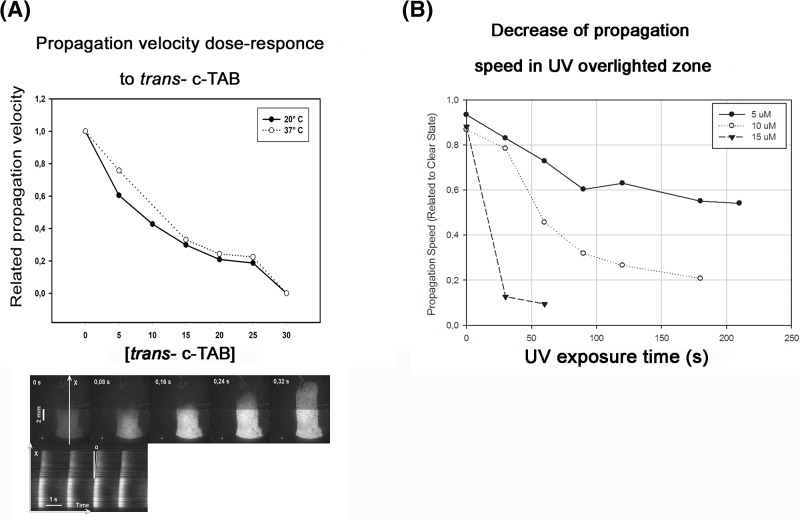
Dose-dependency of speed of the excitation propagation for *trans*- c-TAB at 20 and 37°C (**A**) c-TAB was added to the excitable NRVM monolayer in concentrations of 5–30 µM at 23 and 37°C. It can be seen that the excitation of the NRVM monolayer was suppressed and there is no significant difference in the action of *trans*-c-TAB between 20 and 37°C. Bottom line: Frames of optical mapping movie and time–space plot for *х* line. White lines represent excitation waves. Propagation velocity is calculated as angle cotangent multiplied by dpi/fps ratio. (**B)** Influence of duration of UV exposure on speed of the excitation propagation. The NRVM monolayer in a medium with a constant concentration of c-TAB was exposed to UV light. The propagation speed of the excitation was measured during the illumination. At a concentration of 5 μM, complete suppression was not achieved; at 10 μM, complete suppression was achieved after 3 min of UV exposure; at 15 μM, complete suppression was achieved after 1 min of UV exposure.

Dose-dependent effect of suppression of the excitation waves under *trans*- c-TAB was studied at 20 and 37°C (Figure 5A) (S12_movie–S18_movie in Supplementary Materials) with the aid of optical mapping setup. In the concentration range 5–25 μM propagation velocity monotonically decreased. The complete propagation suppression was obtained at 30 μM. UV-dependent effect of *trans*- c-TAB was demonstrated for the under-suppressive (<30 μM) concentrations (Figure 5B). Propagation velocity in the culture was measured at three concentrations, 5 μM, 10 μM and 15 μM, simultaneously irradiating cell layer with UV. With continuous UV irradiation, it was possible to obtain full suppression of the excitation waves: after 60 s for 15 μM and after 180 s for 10 μM of c-TAB. No complete suppression was obtained for the 5 μM concentration.

### Effect of c-TAB application on action potential and voltage-gated ion channels

Because generation and propagation of the action potential in cardiac cells is based on the activity of voltage-gated ion channels, the modulation of their activity by the application of c-TAB was studied by the patch-clamp method. The whole-cell patch-clamp technique in the current-clamp and voltage-clamp modes was employed to record the action potential (AP) and the currents of the voltage-gated ion channels, respectively.

**Figure 6:**
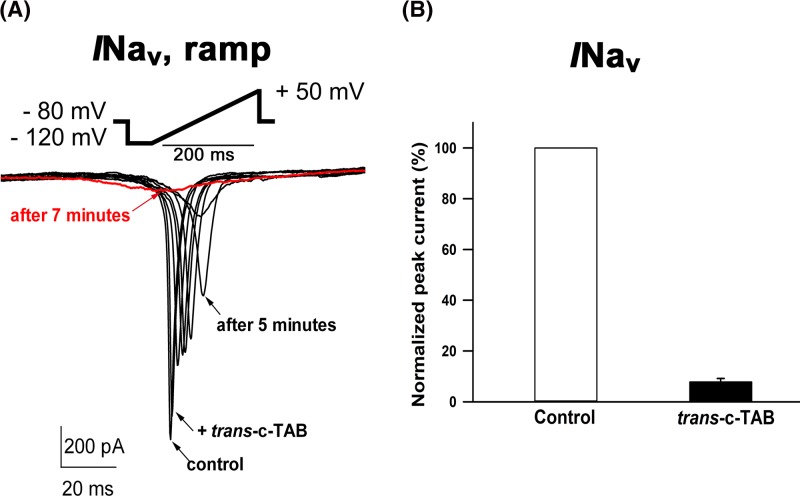
Effect of *trans*- c-TAB on voltage-gated Na+ currents in neonatal rat ventricular myocytes (**A**) Effect of *trans*- c-TAB on action potential (AP) formation in rat neonatal myocytes. Superimposed representative traces of APs recorded within the same rat neonatal myocyte in control conditions and during exposure to 60 μM *trans-* c-TAB. Dynamic of suppression of voltage-gated Na+ current in the presence of *trans*-c-TAB. Scaled ramp-evoked currents recorded in response to the same ramp protocol (from −120 to +50 mV, 200 ms, HP −80 mV) in the control and after the addition of 60 μM* trans-* c-TAB. (**C**) Ion currents recorded before and after incubation with 60 μM *trans-* c-TAB and expressed as percentage. Each cardiomyocyte was incubated in the presence of c-TAB at room temperature for at least 3 min in a measuring chamber. The data represent the means ± SEM, *n*=6, statistical significance was *P*<0.05.

Current pulses of 4-ms duration were applied to elicit the APs. Figure 6A shows the action potential before and after application of the 60 μM c-TAB. It is seen that the form of action potential was changed, upstroke of the AP was decreased after 3-min incubation in the *trans*- c-TAB.

**Figure 7:**
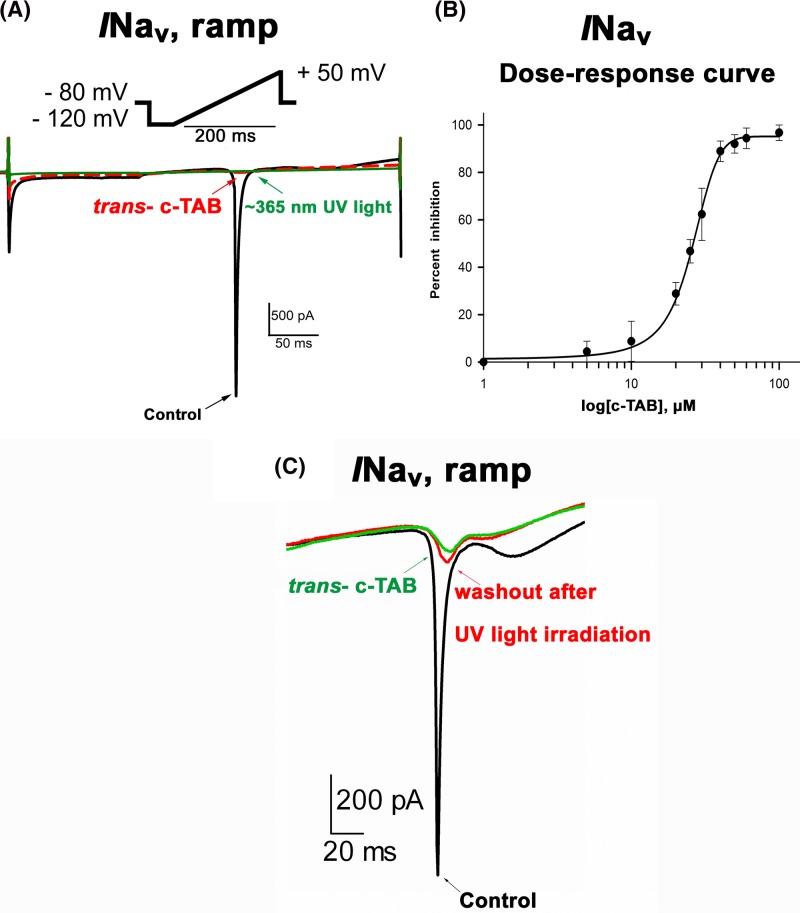
Effect of *trans*-c-TAB and UV-light on voltage-gated Nav current in neonatal rat ventricular myocytes (**A**) Effect of c-TAB on ramp currents in neonatal rat ventricular myocytes. Scaled ramp-evoked currents recorded in response to the same ramp protocol (from −120 to +50 mV, 200 ms) in the control and after the addition of 60 μM* trans*- c-TAB. Three minutes after the application, the current was inhibited by approximately 90% relative to that of the control, and it was not restored after UV irradiation (∼365 nm). Similar results were obtained in more than three additional cells. (**B**) Concentration dependency for *trans*- c-TAB-induced inhibition of INav in neonatal rat ventricular cardiomyocytes. Mean ± SEM, *n*=3–4 for each point, statistical significance was *P*<0.05. (**C**) Suppression of INav by *trans-* c-TAB, then irradiation with UV cells in the presence of *trans-* c-TAB. After UV irradiation, INav cannot be restored by washing cells from c-TAB.

**Figure 8:**
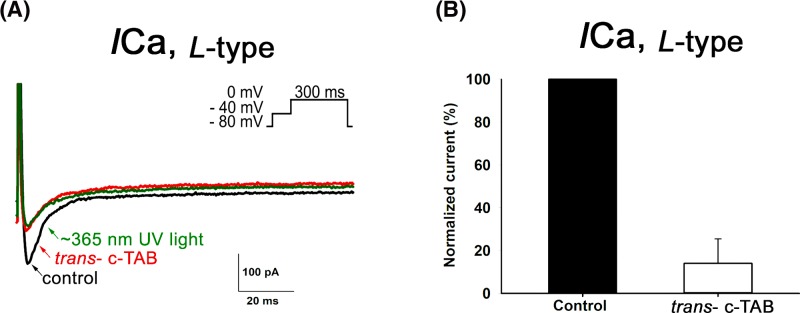
Effect of c-TAB on voltage-gated Ca^2+^ currents in neonatal rat ventricular cardiomyocytes (**A**) L-type Ca^2+^ currents obtained in the absence (control) and presence of 60 μM *trans*- с-TAB and after UV irradiation. Inset: original current trace in response to a voltage step from −40 mV to 0 mV for 300 ms. Inactivation of INav was achieved by a pre-step from a holding potential HP of −80 mV to −40 mV for 100 ms. Similar results were obtained in more than three additional cells. (**B**) Calcium current ICa, L-type recorded before and after treatment with 60 μM с-TAB and expressed as a percentage of the control ICa, L-type*.* Each cardiomyocyte was incubated in the presence of с-TAB at room temperature for ∼3 min in a measuring chamber. The data are the means ± SEM, *n*=5, statistical significance was *P*<0.05.

To detect sodium currents, a voltage step protocol was applied in ramp form, from −120 mV to +50 mV for 200 ms. L-type calcium current was caused by applying the stimulus protocol in step form from −40 mV to 0 mV for 300 ms. It was found that the sodium current and L-type calcium currents in the presence of *trans*- c-TAB in concentrations above 60 μM were suppressed by almost 90% and 80%, respectively (Figures 6–8). It was also found that UV irradiation of cells with *trans*- c-TAB does not lead to the recovery of fast sodium and L-type calcium currents; rather, they remain blocked (Figures 7 and 8). It was not possible to restore them by washing the cells in a c-TAB-free medium (Figure 7C).

Membrane ion currents were restored to ∼80% of the initial value after a thorough washing of the cardiomyocyte cells for 20 min to remove the *trans*- c-TAB (S19_Fig, S20_Fig in Supplementary Materials).

Suppression of the fast sodium current was observed upon addition of the *cis*- form of c-TAB (which was pre-irradiated and then added to the cells) (S19 B_Fig in Supplementary Materials). In these experiments, *trans*- c-TAB was irradiated by UV for 2 min in a container shielded from light and then added to isolated cardiomyocytes. The sodium current was suppressed to 90% (S19 B_Fig in Supplementary Materials). After that, washing the cells for 20 min to remove the *cis-* c-TAB (which was pre-irradiated and then added to the cells) resulted in the recovery of the fast sodium current INav to 70% (S19 B_Fig in Supplementary Materials). In addition, recovery of the sodium current was observed in the cells that were subjected to the *cis-* form of c-TAB, UV irradiated, and then washed out in the c-TAB-free medium (S19 B_Fig in Supplementary Materials).

**Figure 9:**
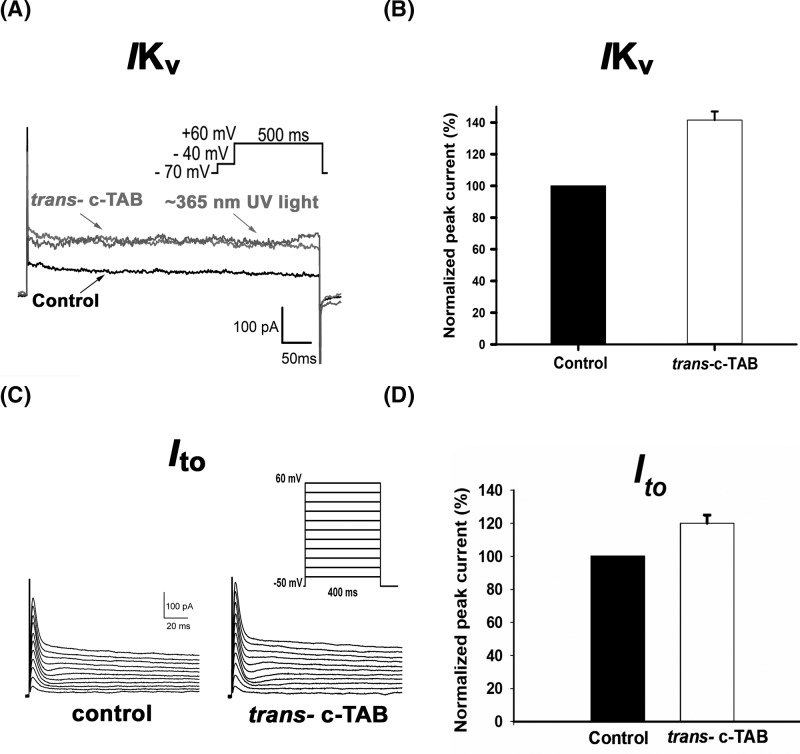
Effect of c-TAB on voltage-gated K+ currents in neonatal rat ventricular cardiomyocytes (**A**) Whole-cell outward K^+^ currents of the control in response to 500 ms depolarizing voltage steps from −70 mV to +60 mV, after application of 60 μM c-TAB under visible light (*trans*-c-TAB) and after ∼365 nm UV irradiation. (**B**) Steady-state K^+^ currents recorded before and after treatment with 60 μM c-TAB and expressed as a percentage of the control IKs. Each cardiomyocyte was incubated in the presence of c-TAB at room temperature for ∼3 min in a measuring chamber. The data are the means ± SEM, *n*=5, statistical significance was *P*<0.05. (**C**) Representative current traces of Ito currents in NRVMs in the control and after application of *trans*- c-TAB. Ito was elicited by depolarizing voltage steps from −50 mV to +60 mV in 10 mV increments. (**D**) Ito currents recorded before and after treatment with 60 μM *trans-*c-TAB and expressed as a percentage of the control Ito. Each cardiomyocyte was incubated in the presence of *trans-* c-TAB at room temperature for ∼3 min in a measuring chamber. The data are the means ± SEM, *n*=4, statistical significance was *P*<0.05.

A slow potassium current was obtained in response to the stimulus protocol in the form of a step from −40 mV to +60 mV for 500 ms. In the case of slow potassium currents IKs in the presence of *trans*- c-TAB, an average increase of 40% was observed (Figure 9A,B). The increased potassium current retained its value due to UV irradiation in the presence of *cis*- c-TAB (Figure 9A). If the UV irradiation was not applied to the cells affected by the c-TAB solution, washing them in the c-TAB-free solution for approximately 20 min resulted in the restoration of the slow potassium current to its control value (S20 B_Fig in Supplementary Materials). A transient potassium current was increased (an average increase of 40% was observed) in the presence of *trans-* c-TAB (Figure 9C).

### ^1H^NMR studies of the interaction of c-TAB with a lipid bilayer

**Figure 10:**
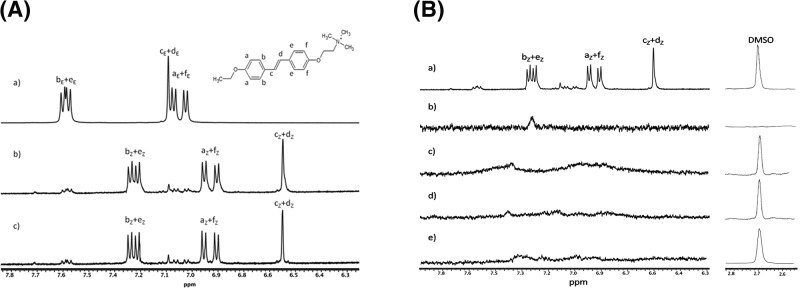
^1^H NMR measurements (**A**) ^1^H NMR spectra of c-TAB in D2O/DMSO-d6 (**a**) c-TAB before UV irradiation; (**b**) c-TAB after UV irradiation; (**c**) c-TAB 24 h after UV irradiation. The ten protons of the stilbene groups are indicated by the letters (a–f). The subscripts E and Z denote *trans*- and *cis*- isomers, respectively. (**B**) ^1^H NMR spectra of c-TAB, lipid bilayer and c-TAB/lipid bilayer mixture in D2O/DMSO-d6 (**a**) c-TAB after UV irradiation; (**b**) lipid bilayer; (**c**) c-TAB/lipid bilayer mixture before UV irradiation; (**d**) c-TAB/lipid bilayer after UV irradiation; (**e**) *cis*- c-TAB/lipid bilayer mixture. The ten protons of the stilbene groups are indicated by the letters (a–f). The subscripts E and Z denote *trans*- and *cis*- isomers, respectively.

The nature of the interaction of c-TAB and the lipid bilayer formed by DOPC molecules (1,2-dioleoyl-sn-glycero-3-phosphocholine) was studied by nuclear magnetic resonance (NMR). Figure 10A shows the signals of aromatic protons in the ^1^H NMR spectra of c-TAB samples before UV irradiation (a), after UV irradiation (b) and a day after UV irradiation (c). Analysis of the spectrum shown in Figure 10A(b) demonstrates that the ratios of *trans-* c-TAB and *cis-* c-TAB isomers after irradiating the sample for 5 min at 365 nm are 8.4% and 91.6%, respectively. A comparison of the spectra in Figure 10A(b) and (c) shows that there is no reverse transition from *cis-* c-TAB to *trans-* c-TAB overnight in the dark.

To study the interaction of a c-TAB molecule with a lipid bilayer, DOPC (1,2-dioleoyl-sn-glycero-3-phosphocholine) was used as a model of a lipid bilayer. Figure 10B shows the signals of aromatic protons in the ^1^H NMR spectra of c-TAB (a); DOPC (b); *E*-c-TAB/DOPC (c); and *Z*-c-TAB/DOPC mixtures (d: *E*-c-TAB was added and irradiated with UV; e: *Z*-c-TAB added to DOPC solution). There are no signals in the DOPC spectrum in the region of aromatic protons. For all samples of c-TAB and DOPC mixtures, the signals of aromatic protons in the ^1^H NMR spectra practically disappear due to significant broadening. The fact that the disappearance of the с-TAB aromatic proton peaks is not due to an insufficient accumulation of the signal is evident from the comparison of the DMSO solvent peaks in the region of 2.7 ppm with signals of aromatic protons. DMSO is present in the solution of c-TAB and, therefore, in the mixtures of c-TAB with DOPC. The ratio of the integral intensities of aromatic signals and DMSO signals is a constant value for all samples, and the amplitude of the signals depends on their width, which leads to the almost complete disappearance of aromatic signals in the spectra of mixtures of c-TAB and DMSO. The broadening of signals of c-TAB molecules is due to a decrease in their mobility due to insertion into the DOPC. Consequently, UV irradiation of C-TAB molecules does not lead to loss of c-TAB and DOPC binding.

## Discussion

We showed in the present study that the stilbene derivative c-TAB, a photosensitizer, is able to reversibly or irreversibly suppress the excitability of cultured cardiac cells. The inhibition effect of c-TAB is independent of its isoform (*cis-* or *trans-*). This inhibition can be reversed by washing the cells in a c-TAB-free medium. However, the inhibition may become permanent if cells incubated in a *trans-* c-TAB-containing medium are irradiated with UV light. Under UV, *trans*–*cis* isomerization of the c-TAB occurs; however, for the inhibition to become “permanent” and irreversible (observed in our experiments in a >48 h period), it is important that this photoisomerization happens in cardiac cells (most probably in the cell membrane). Because cells that have not been exposed to UV can be washed to remove the c-TAB, thus restoring their excitability, projection of the UV light in a pattern assists in obtaining the corresponding pattern of excitability in the cell layer, thus allowing the creation of conducting pathways, obstacles of different shapes, etc, in Supplementary Movies S2, S3 and S4*.* It was also shown that the nature of the excitability blockage in cardiac cells is in the modulation of voltage-gated ion channels. The action potential’s upstroke is decreased and the action potential duration is changed. Under the action of c-TAB in both *trans*- and *cis*- forms, the fast sodium and calcium currents are suppressed, while the slow potassium currents of the repolarization phase increase.

The ^1^H NMR data show that the c-TAB in the *trans*- and *cis-* forms are incorporated into the lipid bilayer. Perhaps the modulation of voltage-gated ion channels occurs as a result of the effect of c-TAB on the cell membrane, which may entail a change in the visco-elastic properties of the membrane. A change in the physical and chemical properties of the lipid bilayer, in turn, can affect the operation of voltage-gated ion channels (VGCs), because voltage sensors are closely connected to the lipid bilayer [18,19]. Thus, the lipid bilayer not only provides the environment for transmembrane protein folding and diffusion, but also actively participates in fine control of protein functionality [18]. Voltage-gated ion channels are also susceptible to stretching [20]. In particular, it is known that a photoinduced phase transition of the lipid bilayer arises due to the inclusion of a small amount of stilbene [21].

As for the prospective applications of the c-TAB, they could be based on the ability of the substance to temporarily turn off the excitation of cardiac cells without destroying them. In cardiology, there are several methods of destroying the regions of cardiac tissue that infringe on the normal propagation of excitation waves, such as unwanted conducting paths and ectopic excitation sources, and sites of re-entry formation. In addition to the surgical removal of the target site of the tissue, methods of catheter ablation with chemical agents, powerful radio frequency signals, laser burning and photochemically using phototoxic substances have been developed [22]. Unfortunately, quite often destroying correct part of the cardiac tissue happens after several trials. One could think about application of c-TAB to the area of the heart under the question, while testing the excitation pattern. If unwanted sources and/or pathways are removed, the suppression of excitation may be done for an extended period of time by fixing it with UV light, or permanently destroying cardiac tissue by conventional methods. Otherwise, it could be possible to wash out c-TAB and go probing another area. We are not discussing here the methods of delivery of the c-TAB solution to the heart tissue *in vivo*, as well as corresponding UV irradiation. Such study should involve whole heart experiments, for example in Langendorf preparation [23]. The ability of c-TAB to preserve the excitability block after transition from *trans-* form to *cis-* by irradiation with UV (*λ* ∼ 365 nm), which is not detrimental to cells, can be used in “soft” photochemical ablation of the heart or smooth muscle tissue [22,24]. Using c-TAB, it is possible to render the pathological part of the heart tissue non-conductive instead of performing the traditional destruction/ablation of cells, which results in scars in the injured zone formed by the connective tissue. Another potential application for c-TAB could be precise anesthesia, since long blocking of voltage-gated sodium channels might be performed within a very small area, limited by the UV focusing spot. The lower toxicity levels compared with azobenzene derivatives and inversion of the effect allows us to look at c-TAB as a potential photosensitizer-blocker, as a local anesthetic for nervous tissue [25] so that locality of anesthesia can be determined by irradiation of a specific area on the human body.

## Conclusion

The present study showed that c-TAB (2- {4- [(E) -2- (4-ethoxyphenyl) vinyl] phenoxy} ethyl) trimethylammonium bromide) can reversibly inhibit excitability of cultured cardiac cells in both *trans-* and *cis-* form. The inhibition is reversed by washing the c-TAB off the cells. It is possible to achieve permanent inhibition of the excitability in the cells if cells, incubated in the solution with the *trans*- form of c-TAB, are irradiated with UV. The excitation blockage, whether temporary or permanent, did not lead to cell death or noticeably damage cardiac cells for at least 72 h.

## Supporting information

**S1_Movie:** 

**S2_Movie:** 

**S3_Movie:** 

**S4_Movie:** 

**S5_Movie:** 

**S6_Movie:** 

**S7_Movie:** 

**S8_Movie:** 

**S9_Movie:** 

**S10_Movie:** 

**S11_Movie:** 

**S12_Movie:** 

**S13_Movie:** 

**S14_Movie:** 

**S15_Movie:** 

**S16_Movie:** 

**S17_Movie:** 

**S18_Movie:** 

**Fig S19:** 

**Fig S20:** 

**Fig S21:** 

**Fig S22:** 

**Supplemental Table S1:** Chemical shifts of *E*-c-TAB molecule in ^1^H and ^13^C spectra

**Supplemental Table S2:** Chemical shifts of *Z*-c-TAB in ^1^H and ^13^C spectra

## Abbreviations

AP: action potential
AzoTAB: azobenzenetrimethylammonium bromide
c-TAB: stilbene trimethylammonium bromide
HP: holding potential
IСаv,*_L_*: current of voltage-gated calcium channel L-type
IKs: current of voltage-gated slow potassium channels
INav: current of voltage-gated fast sodium channels
Ito: current of voltage-gated outward transient channels
UV: ultraviolet
