# Supplementary material for: Stilbene derivative as a photosensitive compound to control the excitability of neonatal rat cardiomyocytes
Source: Biosci Rep. 2019 Jan 25;39(1):BSR20181849. doi: 10.1042/BSR20181849 (PMC6419148; doi:10.1042/BSR20181849)
Supplement: Supplementary file 19 [file bsr-39-bsr20181849_Supp1.pdf]

S1_ movie – S18_ movie are movies from optical mapping:

S1_movie

Control of excitable NRVM monolayer. Excitation waves in clear culture. Border under the sample is a screen to form UV exposure and shadow zones.

S2_movie

Addition of 50 µM of *trans*- c-TAB to control (S1_movie) the excitable NRVM monolayer. The excitation of the NRVM monolayer was suppressed.

S3_movie

Then after addition of the *trans*- c-TAB only the right part of the monolayer (from S2_movie) was irradiated by UV light for one minute. The excitation of the NRVM monolayer was not restored.

S4_movie

Excitability was restored after the c-TAB was washed out of the of NRVM monolayer in the half of the sample that had not been previously illuminated (propagation restored in the left zone after c-TAB wash out from S3_movie)

S5_ movie – S11_ Effect of *cis*- c-TAB on NRVM monolayer: Addition of 30 and 50 µM of cis- c-TAB leads to suppression of the excitation of the NRVM monolayer. Similar results were obtained in three additional samples of NRVM monolayers.

S5_movie

Control of excitable NRVM monolayer. Excitation waves in clear culture

S6_movie

Addition of 30 µM of *cis*- c-TAB leads to suppression of the excitation of the NRVM monolayer.

S7_movie

The excitation of the NRVM monolayer was restored after wash out

S8_movie

Control of excitable NRVM monolayer. Excitation waves in clear culture

S9_movie

Addition of 50 μM of previously UV-exposed solution c-TAB leads to suppression of the excitation of the NRVM monolayer. The excitation supressed at 50 μM *cis*- c-TAB (previously UV-exposed solution and then added to the NRVM)

S10_movie

Additionally irradiated the NRVM by UV-light in the presence of 50 μM *cis*- c-TAB for 4 minutes by UV-light (~365 nm)

S11_movie

The suppressed by *cis*- c-TAB excitation of NRVM (S10_movie) was restored after wash out

S_12 – S_18 - Dose-dependency of speed of the excitation propagation for *trans*- c-TAB 37°C. c-TAB was added to the excitable NRVM monolayer in concentrations of 5–30 μM at 37°C. It can be seen that the excitation of the NRVM monolayer was . The NRVM monolayer in a medium with a constant concentration of c-TAB was exposed to UV light. The propagation speed of the excitation was measured during the illumination. At a concentration of 5 μM, complete suppression was not achieved; at 10 μM, complete suppression was achieved after three minutes of UV exposure; at 15 μM, complete suppression was achieved after one minute of UV exposure.

S12_movie

Control of the excitation waves in clear culture of NRVM at 37°C

S13_movie

Excitation waves after addition 5 μM of *trans*- c-TAB at 37°C

S14_movie

Excitation waves after addition 10 μM of *trans*- c-TAB at 37°C

S15_movie

Excitation waves after addition 15 μM of trans- c-TAB at 37°C

S16_movie

Excitation waves after addition 20 μM of trans- c-TAB at 37°C

S17_movie

Excitation waves after addition 25 μM of trans- c-TAB at 37°C

S18_movie

Excitation waves after addition 30 μM of trans- c-TAB at 37°C

S19 Fig
Restoring INa+ after washout *trans*- c-TAB and *cis*- c-TAB from cells

A. Effect of c-TAB on ramp currents in neonatal rat ventricular myocytes. Scaled ramp-evoked currents recorded in response to the same ramp protocol (from -120 to +50 mV, 200 ms) in the control, after the addition of 60 μM *trans*- c-TAB and washout cells from c-TAB. Three minutes after the application, the current was inhibited by approximately 90% relative to that of the control, and it was restored after washout. Similar results were obtained in more than three additional cells.

B. Effect of c-TAB on ramp currents in neonatal rat ventricular myocytes. Scaled ramp-evoked currents recorded in response to the same ramp protocol (from -120 to +50 mV, 200 ms) in the control, after the addition of 60 μM *cis*- c-TAB and washout cells from c-TAB. Three minutes after the application, the current was inhibited by approximately 90% relative to that of the control, and it was restored after washout. Similar results were obtained in more than three additional cells.

S20 Fig
Restoring ICa, L-type and IK+ after washout cells from *trans*- c-TAB

A. L-type Ca2+ currents obtained in the absence (control), presence of 60 μM *trans*-c-TAB and after washout cells from c-TAB. Inactivation of INav was achieved by a pre-step from a holding potential HP of -80 mV to -40 mV for 100 ms. Similar results were obtained in more than three additional cells.

B. Whole-cell outward K+ currents of the control in response to 500 ms depolarizing voltage steps from -70 mV to +60 mV, after application of 60 μM c-TAB and after washout cells from c-TAB.

S21
NMR analyze of *trans*-, *cis*- c-TAB and trans-, *cis*- c-TAB with lipids DOPC

S22 Fig
General scheme for the synthesis of c-TAB (source is patent: MIPT, RU Pat., RU 2515502 C1, 2012).

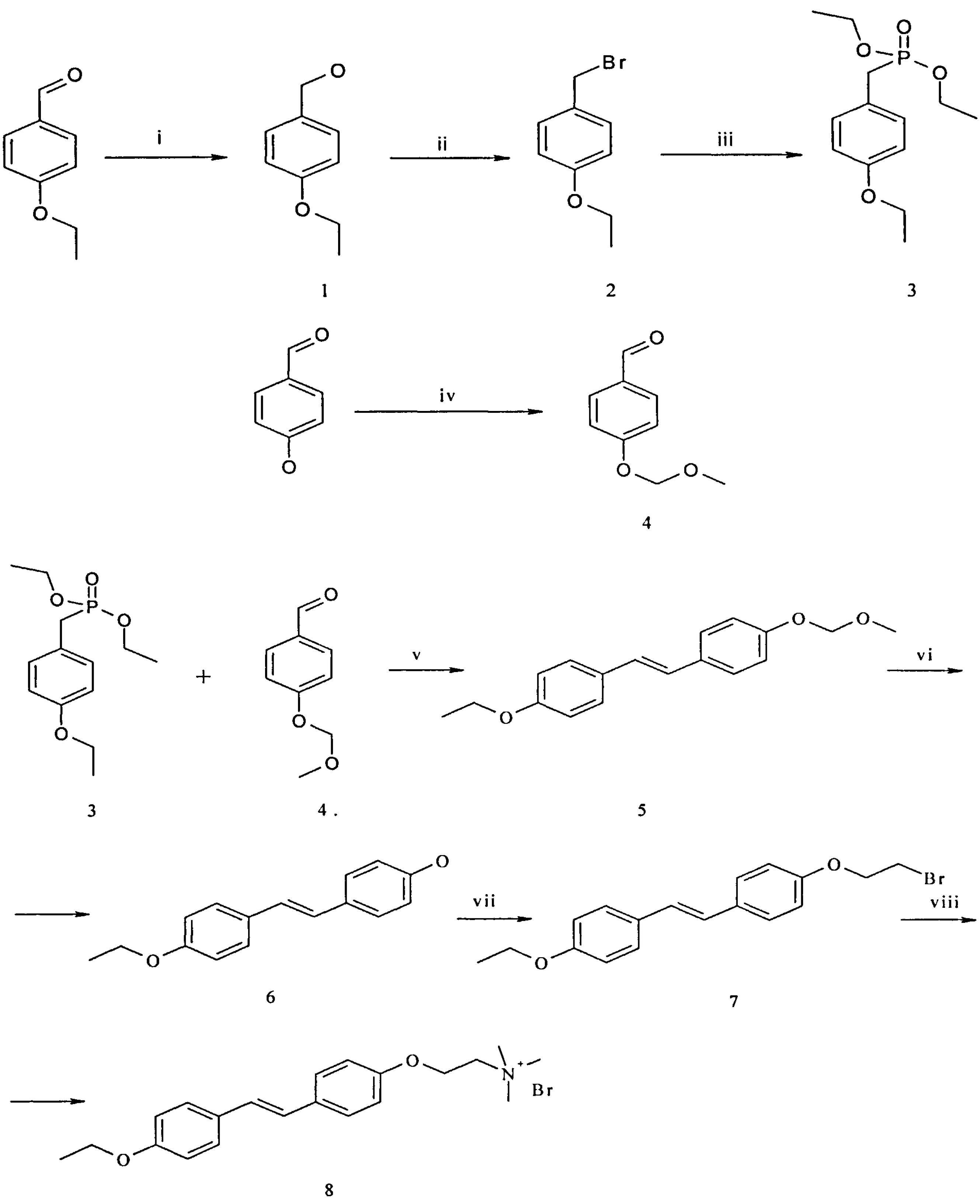

General scheme for the synthesis (2- {4- [(E) -2- (4-ethoxyphenyl) vinyl] phenoxy} ethyl) trimethylammonium bromide)

^{1}H-NMR Spectrum *E*-c-TAB:

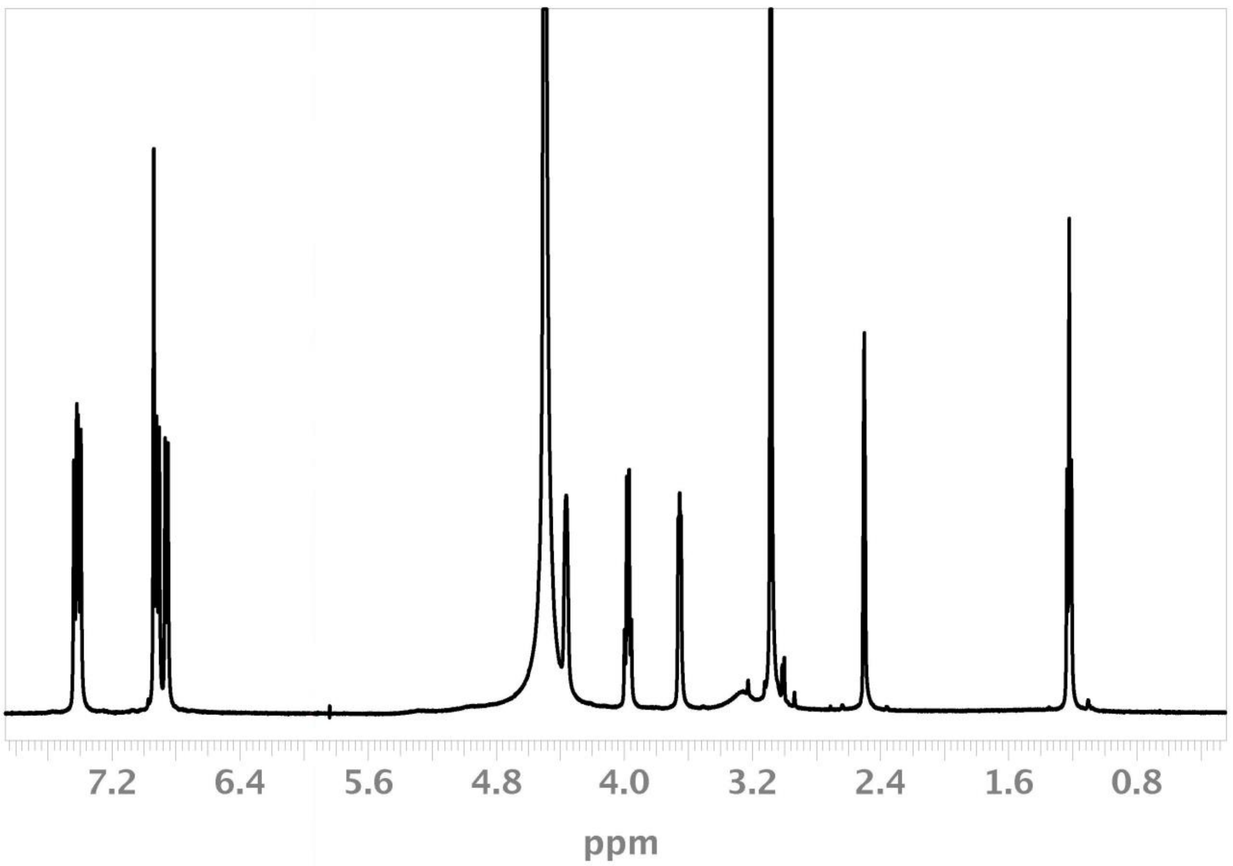

^{13}C-NMR spectrum of *E*-c-TAB:

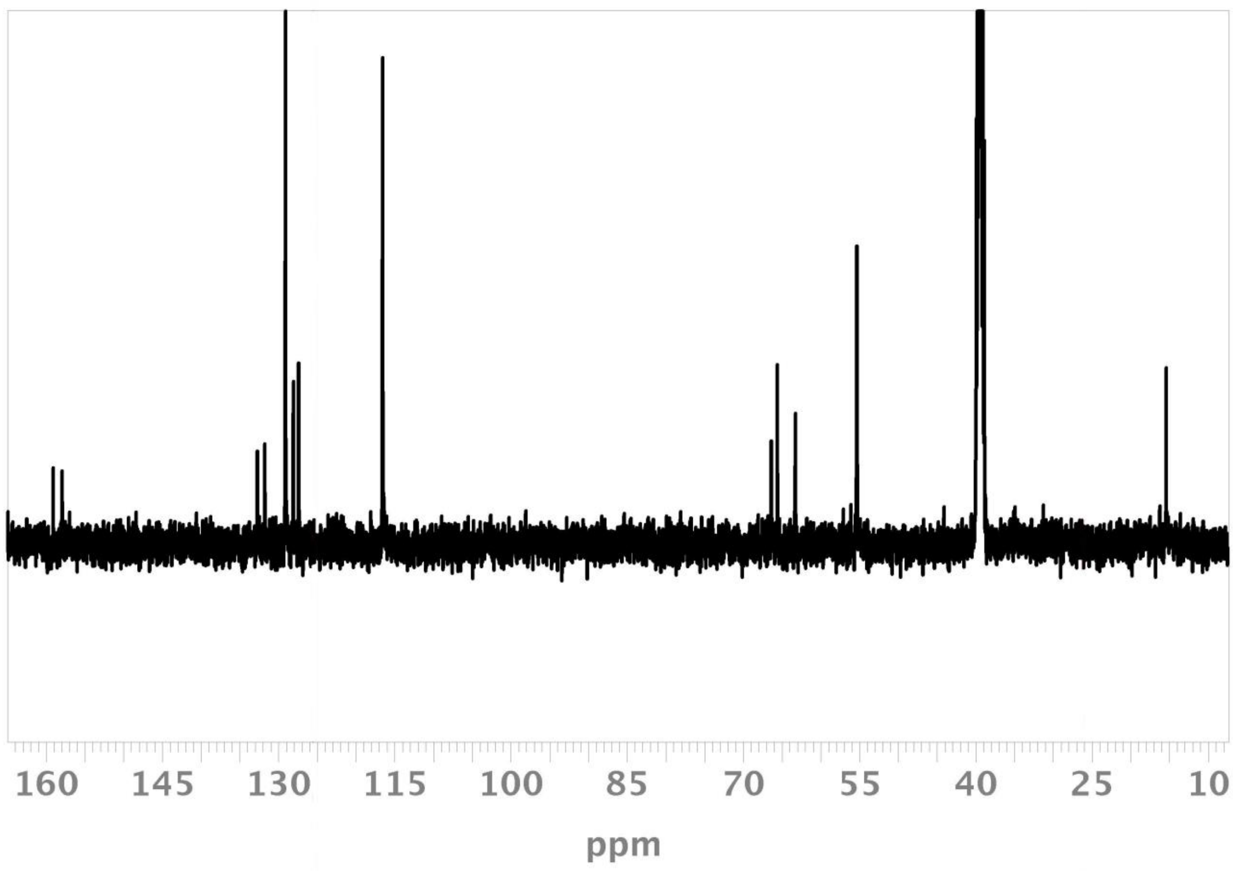

^{1}H-^{1}H COSY spectrum *E*-c-TAB:

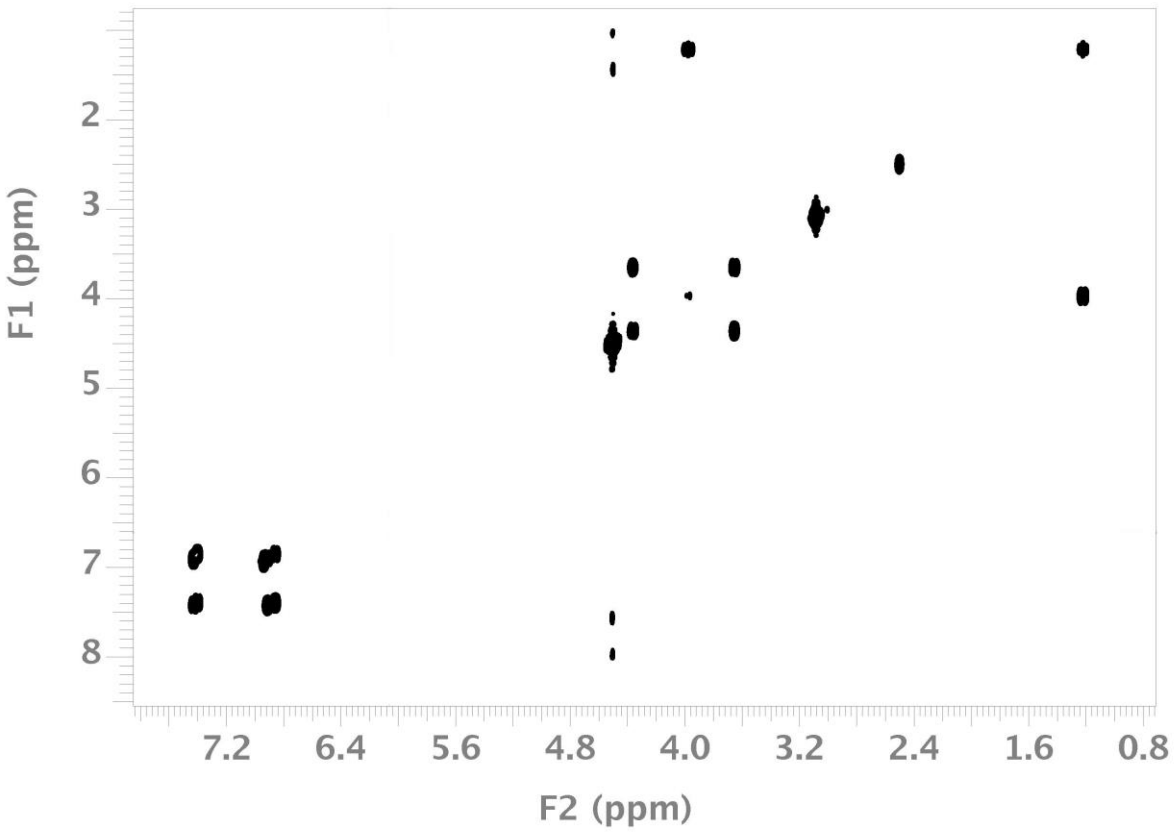

HMBC spectrum *E*-c-TAB:

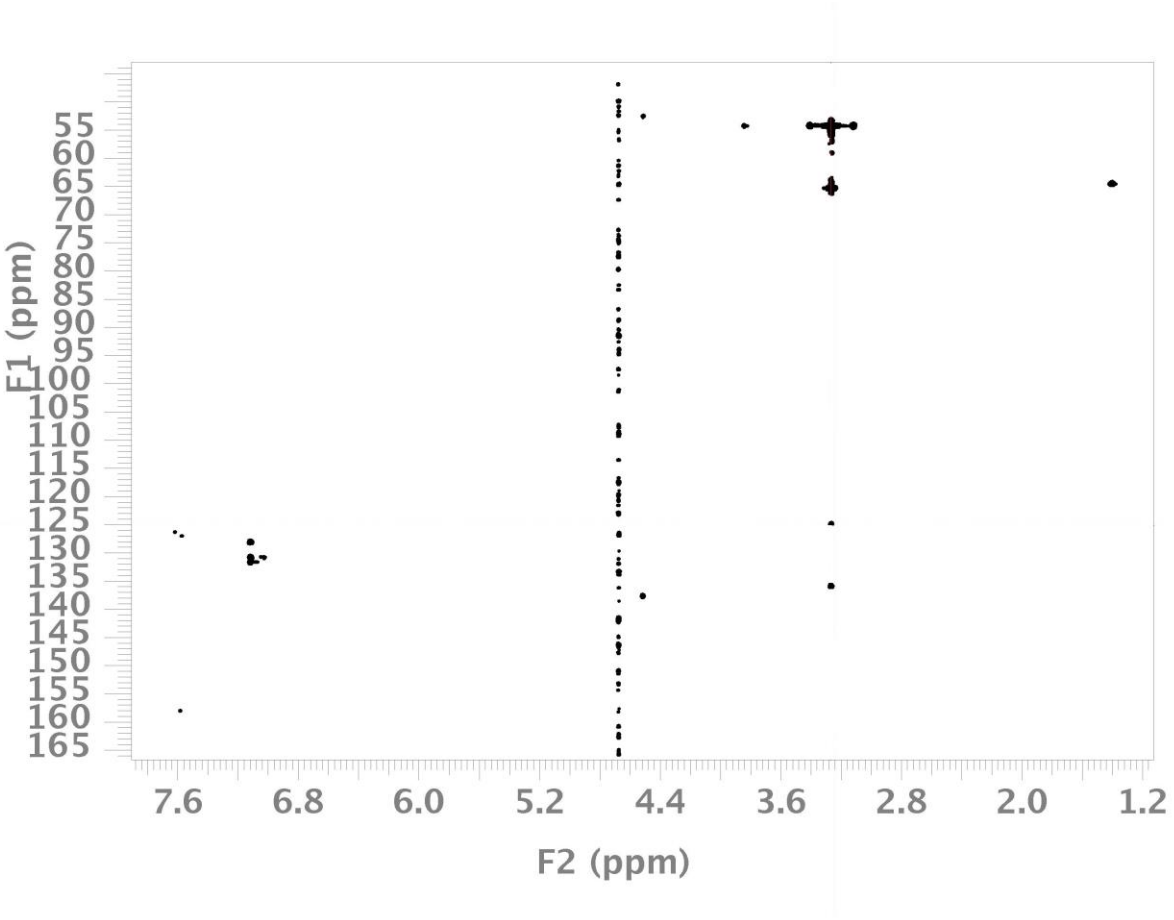

HSQC spectrum *E*-c-TAB:

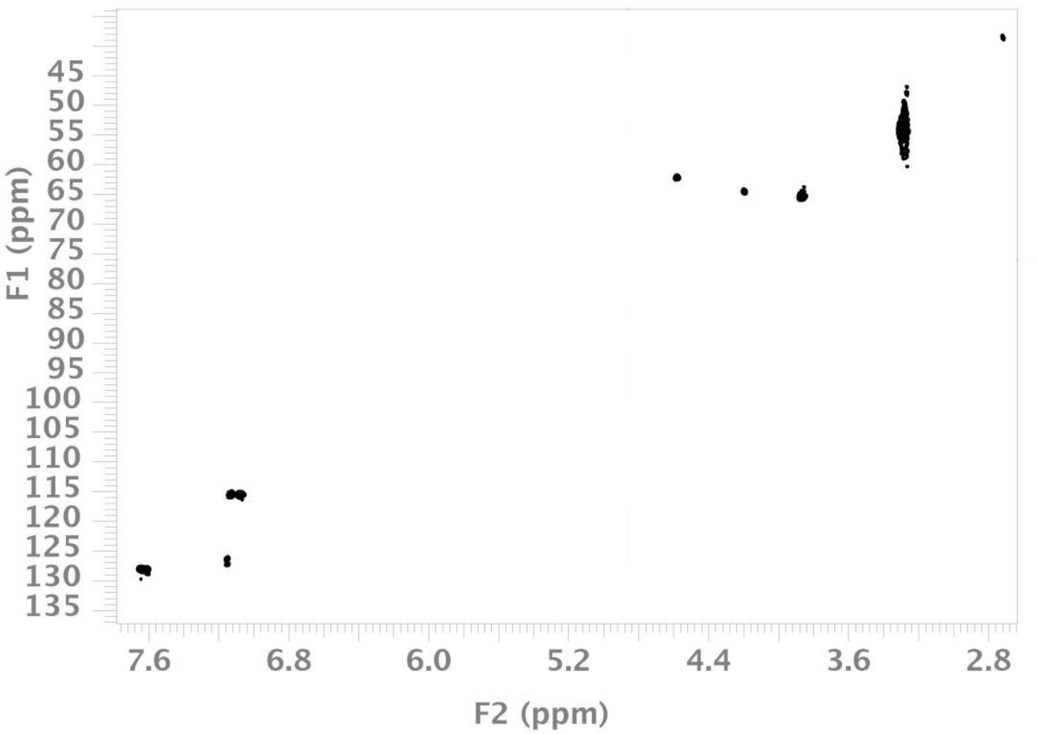

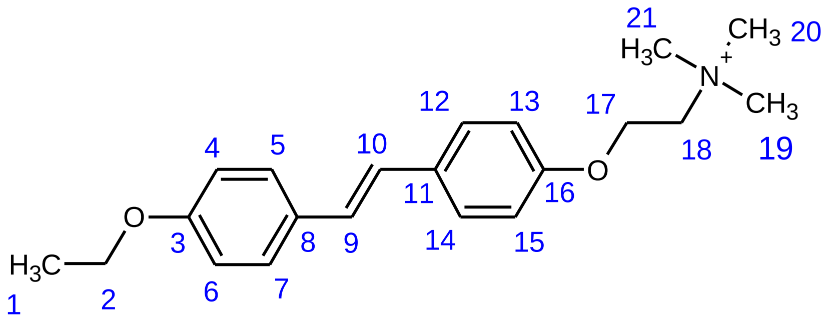

The molecule of *E*-c-TAB

Spectrum characteristics of *E*-c-TAB (Mixture DMSO-d6/D2O calibration for solvent (δ=2,71 м.д. (1H) и 39,39 (13C)), t=27º)

Table. Chemical shifts of *E*-c-TAB molecule in ^{1}H and ^{13}C spectra

№ of atom	Chemical shift		№ of atom	Chemical shift	
	^{1}H	^{13}C		^{1}H	^{13}C
1	1,43 т	15,40	11	—	131,80
2	4,19 кв	65,61	12, 14	7,07 д	116,56
3	—	157,90	13, 15	7,64 д	129,10
4, 6	7,64 д	129,10	16	—	159,04
5, 7	7,07 д	116,56	17	4,57 т	63,28
8	—	132,71	18	3,86 т	66,39
9	7,15 с	127,40	19, 20, 21	3,29 с	55,33
10	7,15 с	128,11			

^{13}C-NMR spectrum of Z-c-TAB:

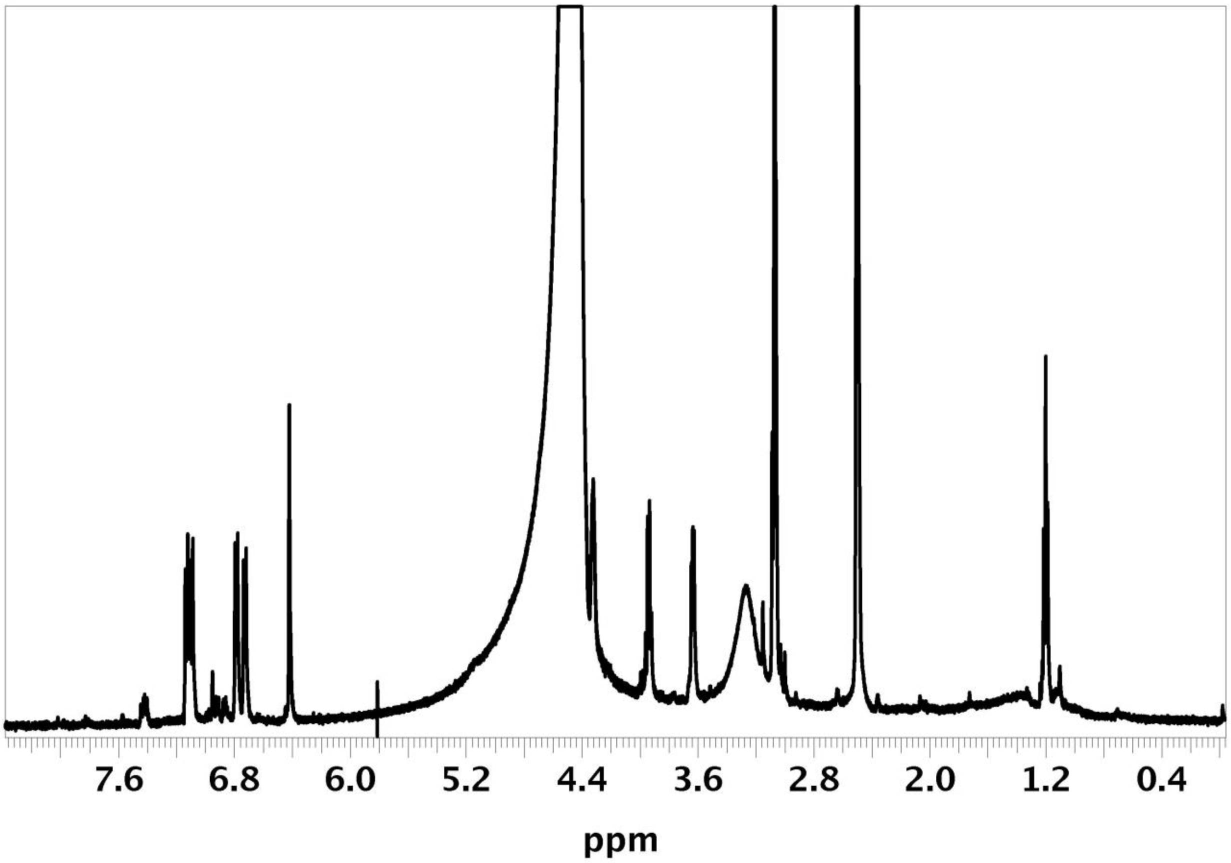

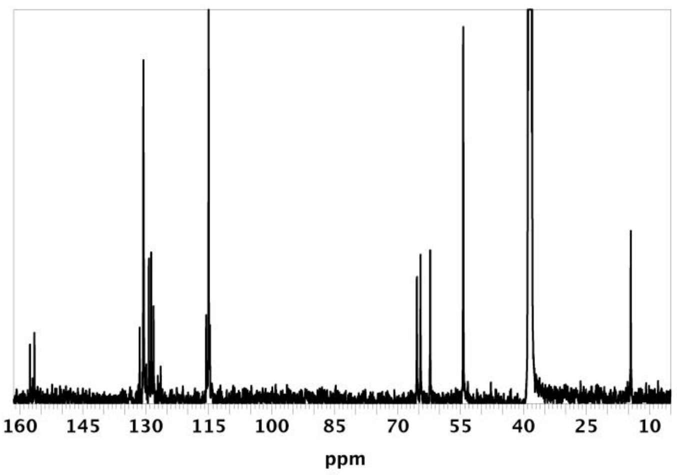

^1H-^1H COSY spectrum of *Z*-c-TAB

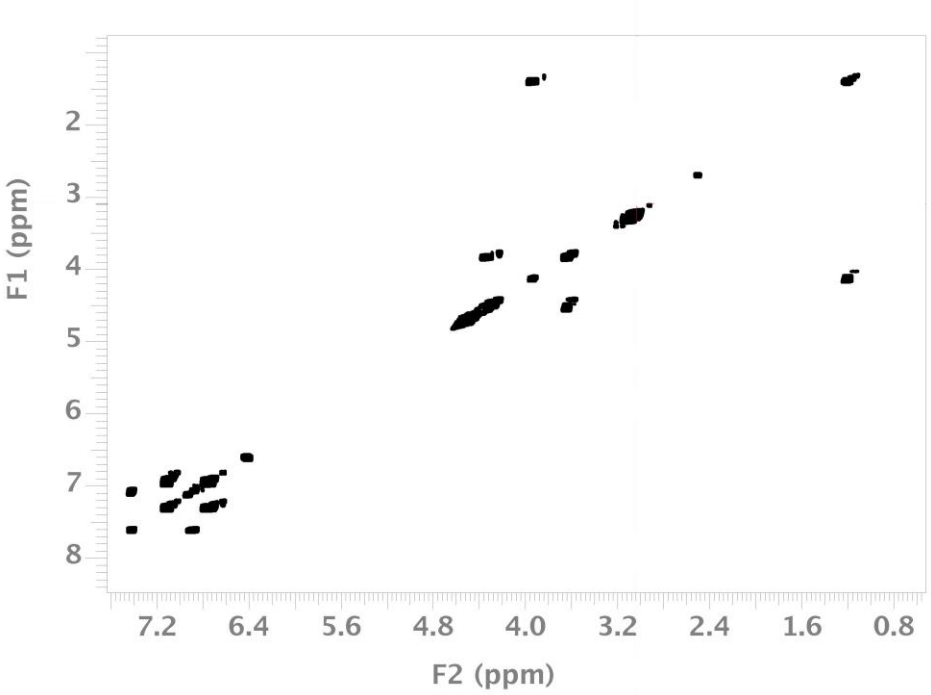

HSQC spectrum of Z-c-TAB:

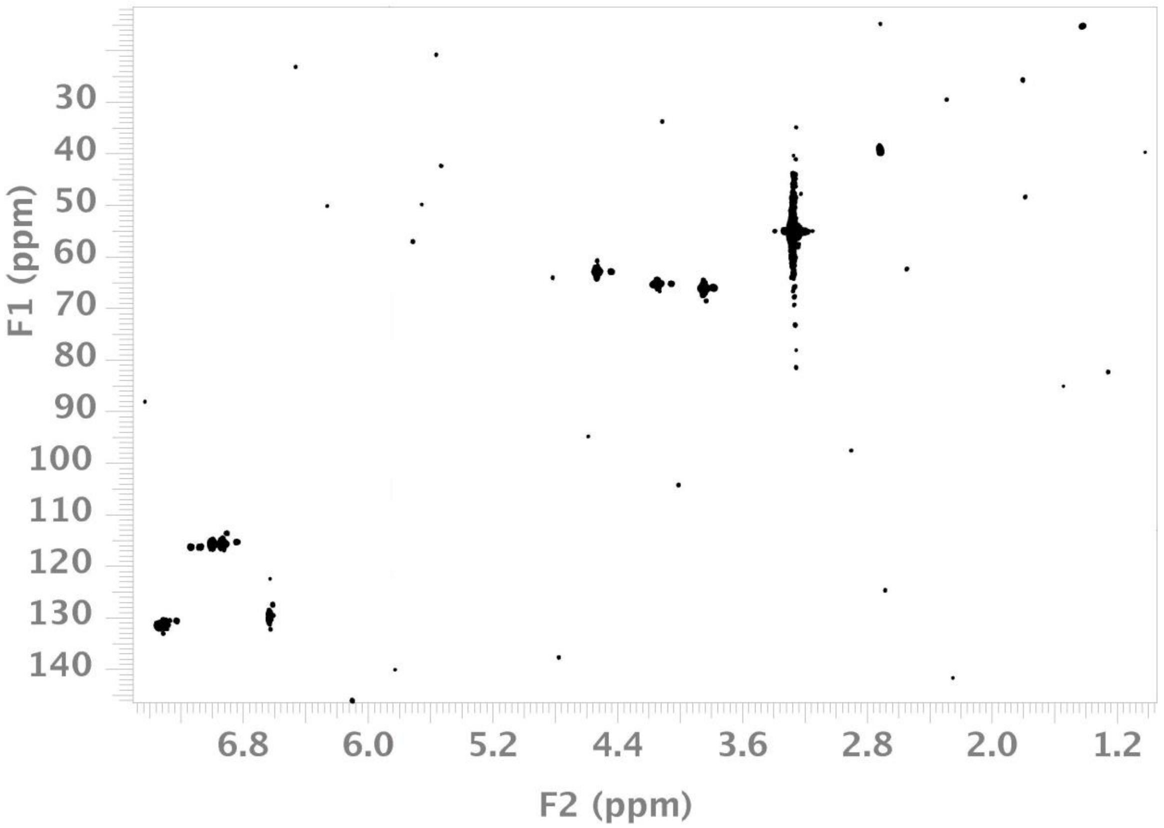

HMBC spectrum of *E*-c-TAB:

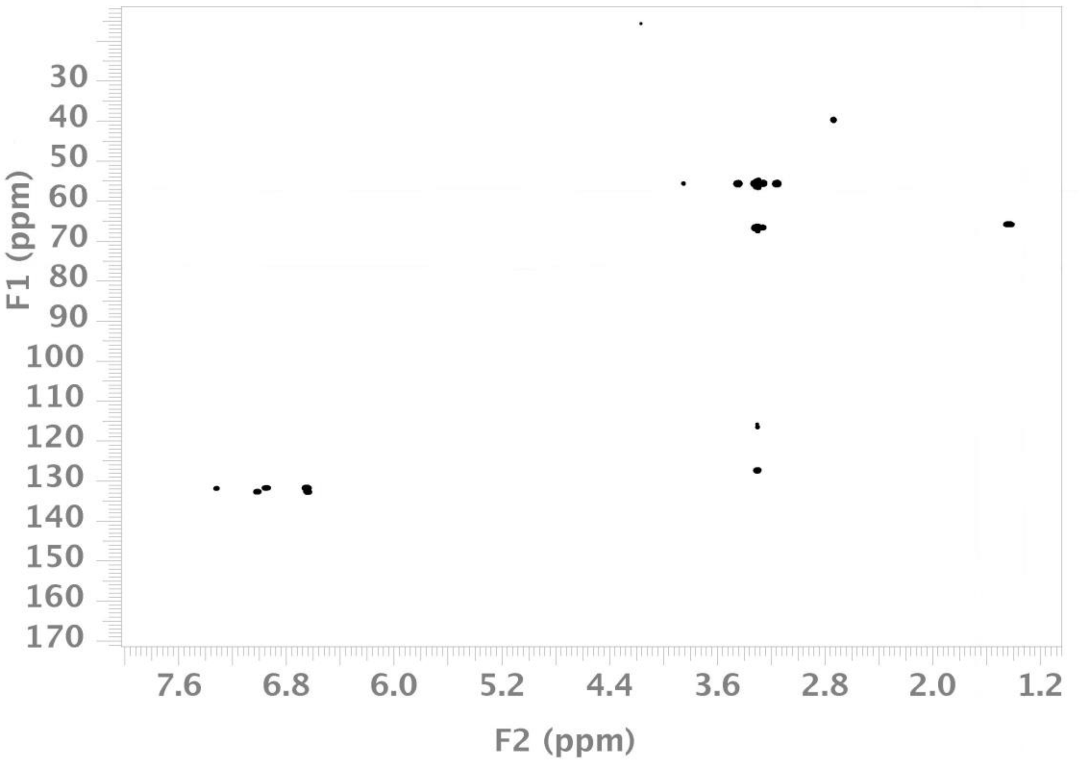

Molecule of *Z*-c-TAB:

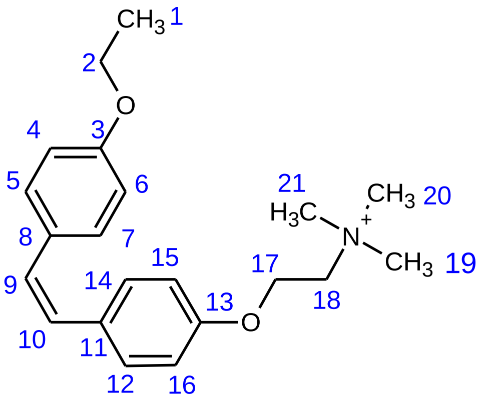

Table. Chemical shifts of *Z*-c-TAB in ^{1}H and ^{13}C spectra

№ of atom	Chemical shift		№ of atom	Chemical shift	
	^{1}H	^{13}C		^{1}H	^{13}C
1	1,40 т	15,4	11	—	131,49
2	4,14кв	65,48	12, 14	6,92 д	115,97
3	—	157,50	13, 15	7,23 д	131,5
4, 6	7,33 д	131,51	16	—	158,54
5, 7	6,99 д	115,97	17	4,52 т	63,18
8	—	132,50	18	3,84 т	66,35
9	6,62 с	130,21	19, 20, 21	3,28 с	55,33
10	6,62 с	129,60			

DOPC+*E*-c-TAB:

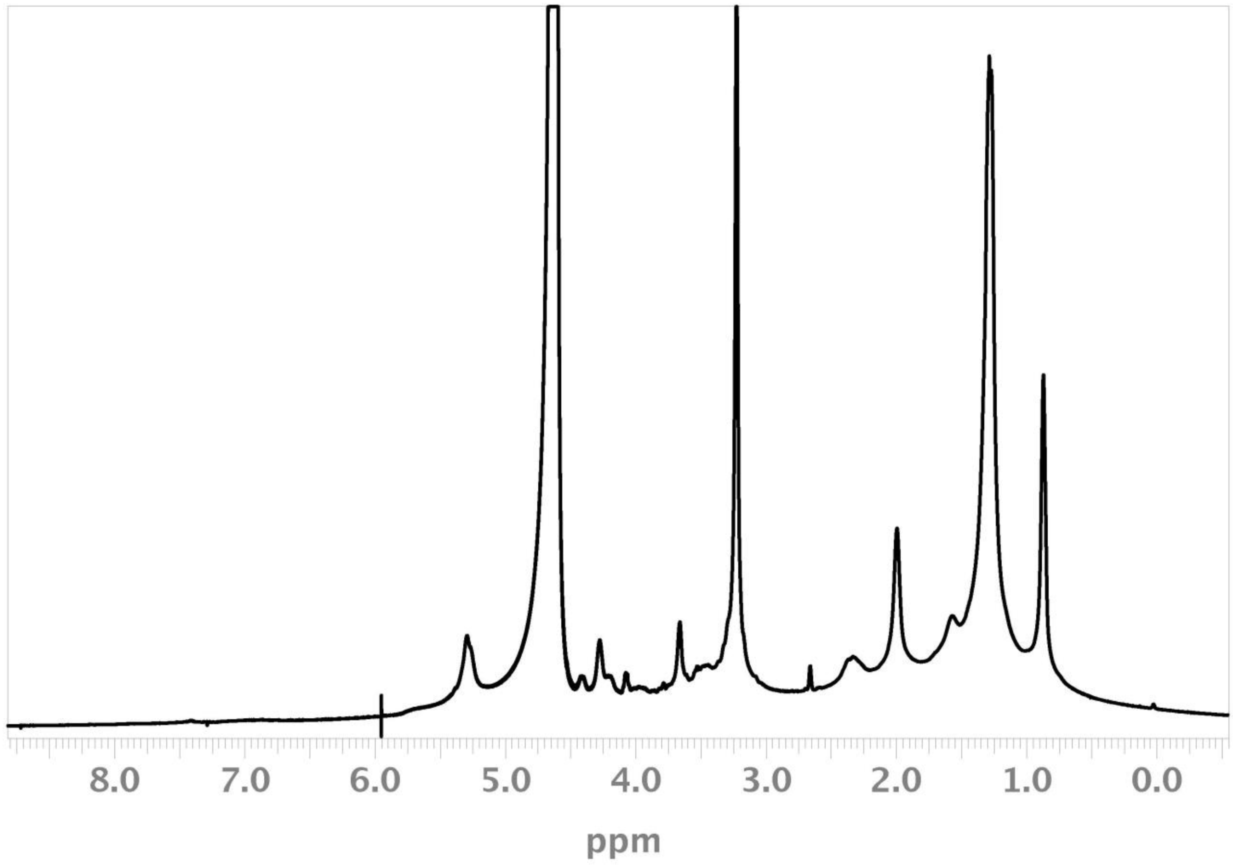

DOPC+*Z*-c-TAB:

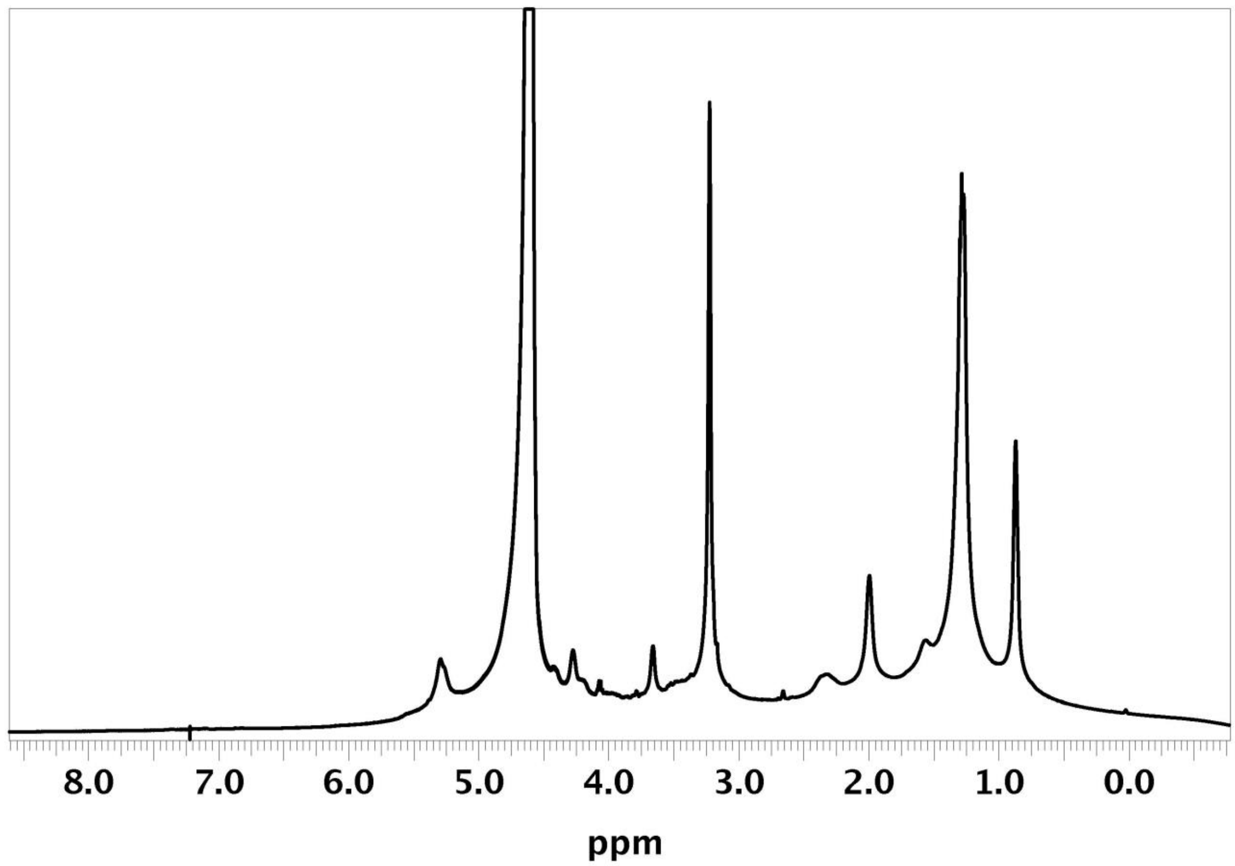

DOPC:

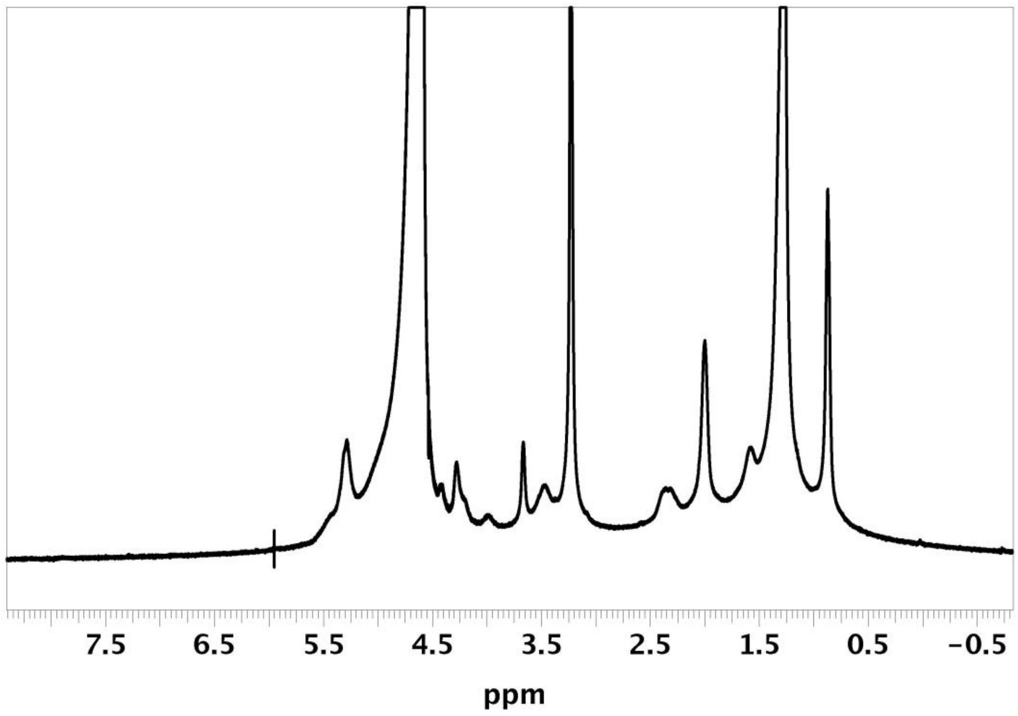

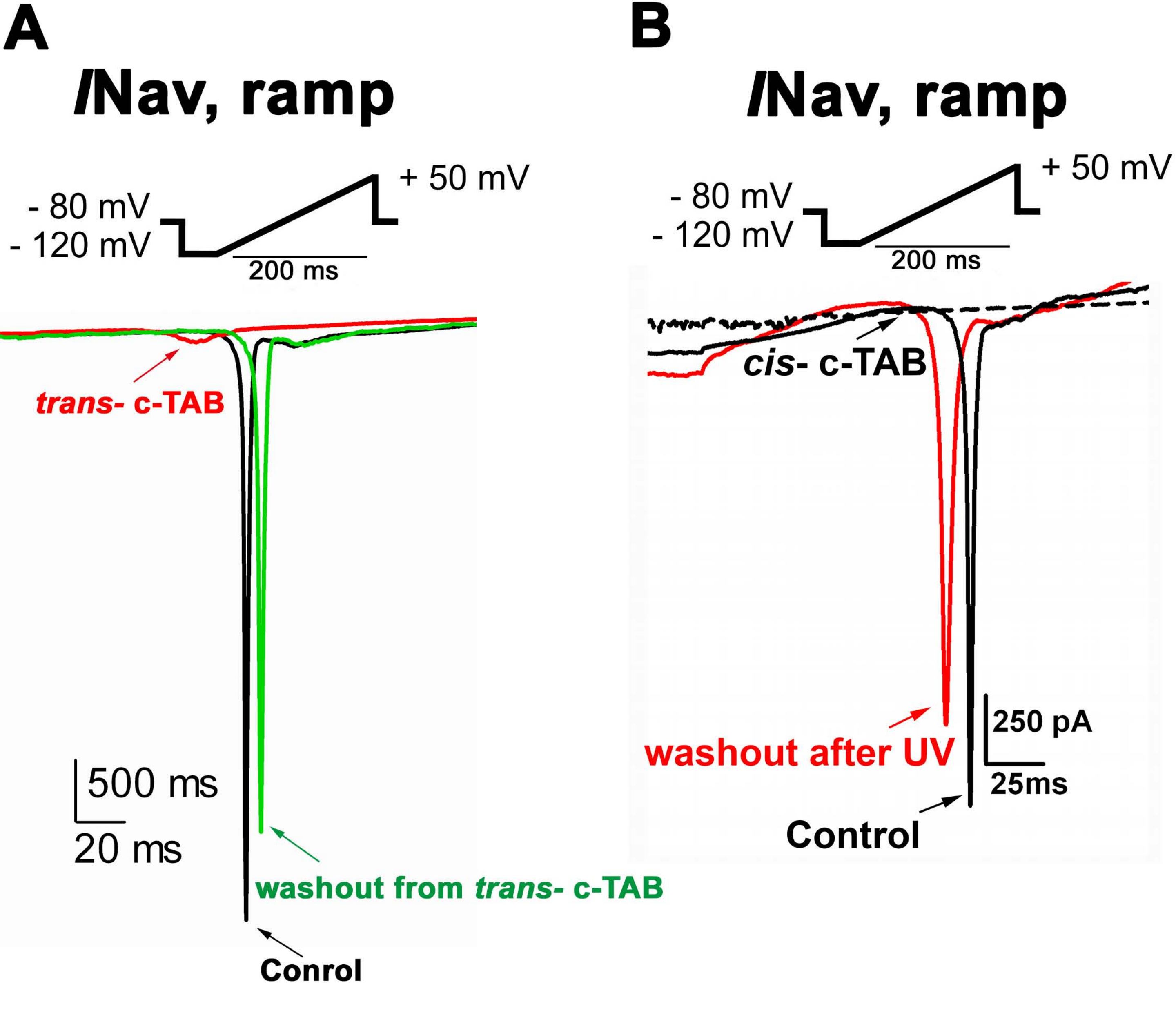
A
INav, ramp
- 80 mV
- 120 mV
+ 50 mV
200 ms
trans- c-TAB
500 ms
20 ms
washout from trans- c-TAB
Conrol
B
INav, ramp
- 80 mV
- 120 mV
+ 50 mV
200 ms
cis- c-TAB
250 pA
25ms
washout after UV
Control

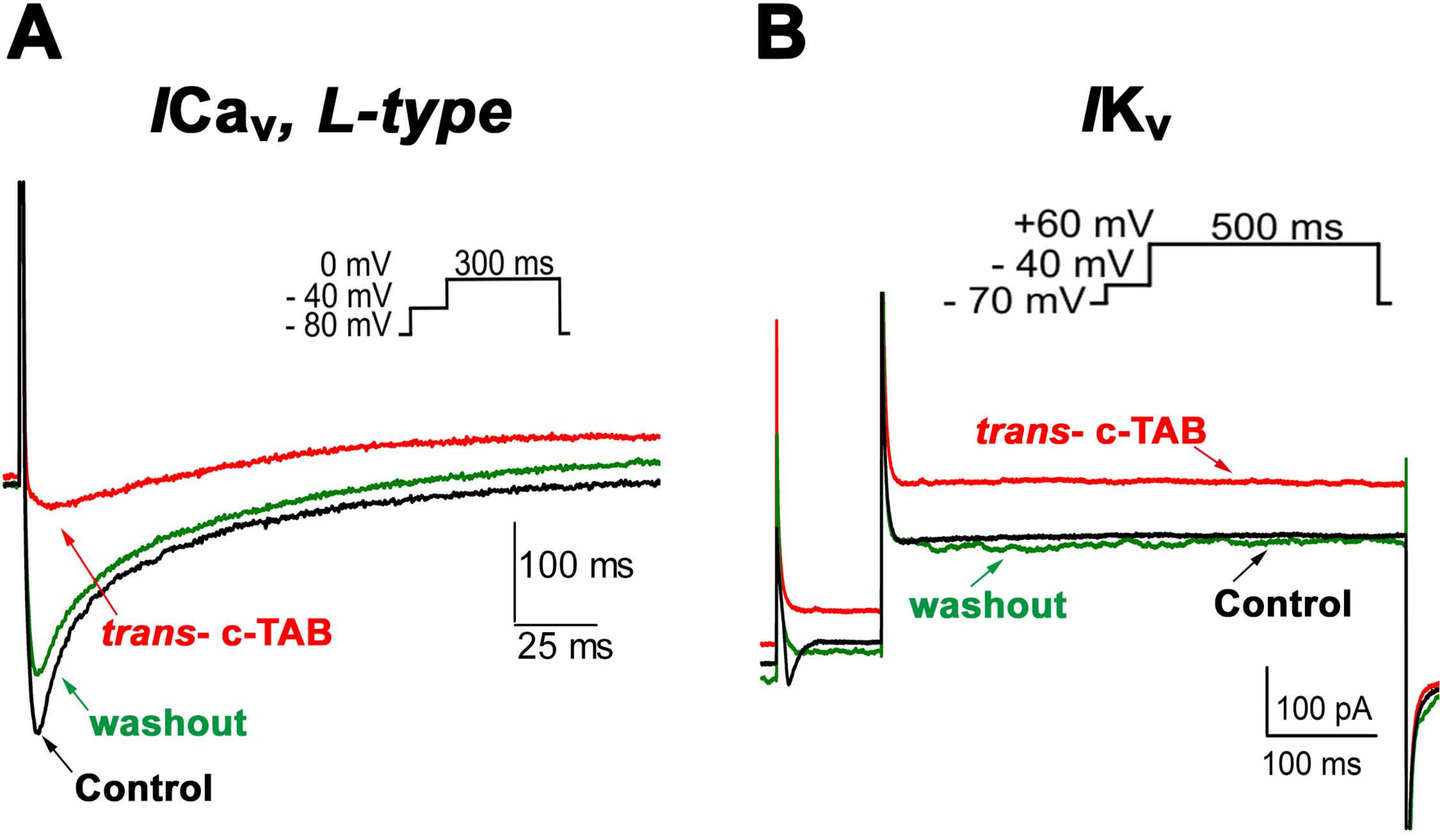
A
ICav, L-type
0 mV
300 ms
- 40 mV
- 80 mV
100 ms
25 ms
trans- c-TAB
washout
Control
B
IKv
+60 mV
500 ms
- 40 mV
- 70 mV
trans- c-TAB
washout
Control
100 pA
100 ms
